# Deep transfer learning approach for automated cell death classification reveals novel ferroptosis-inducing agents in subsets of B-ALL

**DOI:** 10.1038/s41419-025-07704-y

**Published:** 2025-05-18

**Authors:** Paweł Stachura, Zhe Lu, Raphael M. Kronberg, Haifeng C. Xu, Wei Liu, Jia-Wey Tu, Katerina Schaal, Ersen Kameri, Daniel Picard, Silvia von Karstedt, Ute Fischer, Sanil Bhatia, Philipp A. Lang, Arndt Borkhardt, Aleksandra A. Pandyra

**Affiliations:** 1 Department of Pediatric Oncology, Hematology and Clinical Immunology, Medical Faculty, Heinrich-Heine-University, Moorenstrasse 5, 40225 Düsseldorf, Germany; 2 Department of Molecular Medicine II, Medical Faculty, Heinrich-Heine-University, Universitätsstraße 1, 40225 Düsseldorf, Germany; 3 Center for Integrated Oncology Aachen Bonn Cologne Düsseldorf (CIO ABCD), Düsseldorf, Germany; 4 German Consortium for Translational Cancer Research (DKTK), partner site Essen/Düsseldorf, Düsseldorf, Germany; 5 Mathematical Modelling of Biological Systems, Heinrich Heine University, Düsseldorf, North Rhine-Westphalia Germany; 6 Deep-Sea Ecology and Technology, Alfred Wegener Institute Helmholtz Centre for Polar and Marine Research, Bremerhaven, Germany; 7 Institute of Clinical Chemistry and Clinical Pharmacology, University Hospital Bonn, Venusberg-Campus 1, 53127 Bonn, Germany; 8 German Center for Infection Research (DZIF), Partner Site Bonn-Cologne, Bonn, Germany; 9 Cancer Prevention Graduate School (CPGS), German Cancer Research Center (DKFZ), Heidelberg, Germany; 10 Division of Pediatric Neuro-Oncogenomics, German Cancer Research Center (DKFZ), Heidelberg, Germany; 11 Department of Translational Genomics, Faculty of Medicine and University Hospital Cologne, University of Cologne, Weyertal 115b, Cologne, 50931 Germany; 12 CECAD Cluster of Excellence, Faculty of Medicine and University Hospital Cologne, University of Cologne, Joseph-Stelzmann-Straße 26, Cologne, 50931 Germany; 13 Center for Molecular Medicine Cologne, Faculty of Medicine and University Hospital Cologne, University of Cologne, Robert-Koch-Straße 21, Cologne, 50931 Germany

**Keywords:** Acute lymphocytic leukaemia, High-throughput screening, Acute myeloid leukaemia, Drug development, Cell death

## Abstract

Ferroptosis is a recently described type of regulated necrotic cell death whose induction has anti-cancer therapeutic potential, especially in hematological malignancies. However, efforts to uncover novel ferroptosis-inducing therapeutics have been largely unsuccessful. In the current investigation, we classified brightfield microscopy images of tumor cells undergoing defined modes of cell death using deep transfer learning (DTL). The trained DTL network was subsequently combined with high-throughput pharmacological screening approaches using automated live cell imaging to identify novel ferroptosis-inducing functions of the polo-like kinase inhibitor volasertib. Secondary validation showed that subsets of B-cell acute lymphoblastic leukemia (B-ALL) cell lines, namely 697, NALM6, HAL01, REH and primary patient B-ALL samples were sensitive to ferroptosis induction by volasertib. This was accompanied by an upregulation of ferroptosis-related genes post-volasertib treatment in cell lines and patient samples. Importantly, using several leukemia models, we determined that volasertib delayed tumor growth and induced ferroptosis in vivo. Taken together, we have applied DTL to automated live-cell imaging in pharmacological screening to identify novel ferroptosis-inducing functions of a clinically relevant anti-cancer therapeutic.

## Introduction

Cell death is central to homeostatic physiological processes and its dysregulation is linked to several human diseases. The ability to effectively assess cell death is crucial to many assays used in drug development, particularly for the purpose of uncovering novel anti-cancer therapies. There exists a wide plethora of assays scoring cell death parameters and this is accompanied by increasingly specific and novel classifications [1]. Amongst the most expansively-characterized and to some extent interconnected modes of regulated cell death (RCD) that rely on specific molecular machinery are apoptosis, necroptosis, ferroptosis and autophagy. Both intrinsic and extrinsic apoptotic stimuli eventually converge on the executioner protease caspase 3 (CASP3) [2–5] but otherwise follow different biochemical and molecular upstream sequence of events. Following perturbations such as endoplasmic reticulum stress, intrinsic apoptosis is driven by mitochondrial outer membrane permeabilization (MOMP) [1] mediated by members of the BCL2 apoptosis regulator (BCL2) protein family [6]. Extrinsic apoptosis is triggered by binding of ligands (such as FAS ligand or TRAIL) to death receptors like those of the TNF receptor superfamily and downstream caspase-8 activation [7] following its binding to the adapter Fas associated via death domain (FADD) at the death-inducing signalling complex (DISC) [8, 9]. Necroptosis is dependent on RIPK1 and/or 3 activation and mixed lineage kinase domain such as pseudokinase (MLKL) following engagement of death receptors or toll-like receptors (TLRs) [10, 11]. Ferroptosis is caspase-independent [12] and occurs as a result of excess accumulation of iron-dependent lipid reactive oxygen species (lipid ROS) and ensuing lipid peroxidation controlled by the glutathione (GSH)-dependent enzyme glutathione peroxidase 4 (GPX4) [13]. Autophagy is a cellular degradation pathway that clears damaged proteins and organelles. Autophagy does not directly cause cell death, rather RCD might occur when autophagy is blocked as autophagic responses mediate adaptive stress responses and are often cytoprotective. Perturbations that block or stimulate autophagy have an impact on RCD especially in pathophysiological settings [14]. Notably, various modes of regulated cell death differ in their capacity to elicit an inflammatory response which will ultimately impact their therapeutic efficacy. While the cell death types described above are thought to be an important feature of tissue homeostasis, manipulating these modes of cell death in tumor cells where RCD is dysregulated will likely have therapeutic implications for tumor clearance as well as immune system stimulation.

The number of assays evaluating cell death is vast and based on several different technique modalities [15, 16]. Microscopic (fluorescent cyto/histo chemistry, electron, light), fluorescence activated cell sorting (FACS)-based, luminometric, immunoblotting, spectrophotometric (calorimetry/fluorescence based mainly metabolic assays) methods are available with varying degrees of discernability in identifying different modes of cell death. Recently, the application of artificial intelligence (machine and deep learning inclusive) has expanded into the medical field. In radiology, for instance, CT, MRI and X-Rays images are analyzed using deep learning approaches to segment brain tumors [17, 18] or to identify pneumonia in chest X-rays [19]. An automated deep learning method was used to quantitatively evaluate lung burden changes in patients with coronavirus disease using serial CT scans [20]. An image-based environment for capturing, managing, and interpreting pathological information is emerging [21–25]. In experimental models, deep learning was able to predict cell death after one hour of exposure to camptothecin [26]. Others have developed an algorithm to predict neuronal cell death using a robotic microscopy pipeline [27]. We have recently implemented a fast deep learning-based method for classification of patched brightfield images of SARS-CoV-2 infected Vero cells [28]. Here, we applied this methodology and trained a neuronal network to classify several modes of cell death using brightfield microscopy images. Following validation of our neural network, we applied this methodology to a drug screening platform to identify novel death-inducing functions of several drugs which we validated in additional tumor types. Specifically, we found that the polo-like kinase inhibitor volasertib induced ferroptosis in subsets of B-cell acute lymphoblastic leukemia (B-ALL) cell lines, primary patient samples, and in vivo models of leukemia.

## Results

### Several modes of cell death are induced in L929 cells

**Fig. 1:**
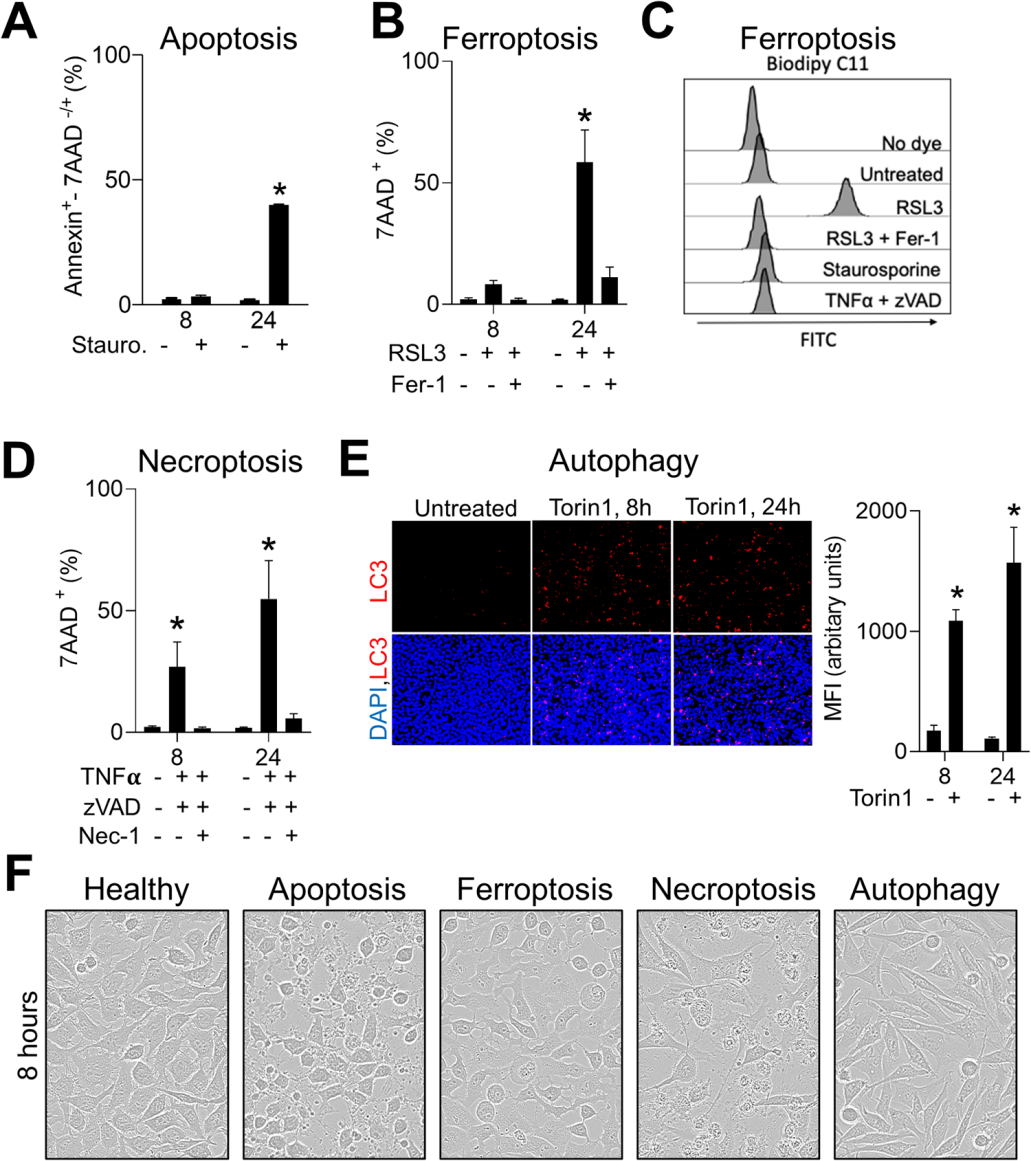
Different modes of cell death can be induced in L929 cells. **A**, **B** L929 cells were analyzed for 7AAD and/or Annexin V (**A**) positivity using FACS after 8 or 24 hour treatment with (**A**) staurosporine and (**B**) RSL3 and Fer-1 (*n* = 4). **C** L929 cells were treated as indicated with the same concentrations as in (**A**), (**B**), (**D**), stained with BODIPY C11 and FACS analyzed in the FITC channel for ferroptosis induction (*n* = 4, a representative histogram is shown). **D** L929 cells were analyzed for 7AAD positivity after 8 and 24-hour treatment with zVAD, TNFα and Nec-1 (*n* = 4). (**E** left panel) L929 cells were treated with Torin1 for 8 or 24 hours and evaluated for LC3 and DAPI using immunofluorescence (representative images of n of 4 are shown). LC3 fluorescent signal was quantified in the right panel. **F** Representative brightfield images of L929 cells after 8 hour treatment with staurosporine for apoptosis induction, RSL3 for ferroptosis induction, zVAD and TNFα for necroptosis induction and Torin1 for autophagy induction are shown (representative images of n of 3 are shown). In every experiment, the following concentrations were used: staurosporine (1 µM), zVAD (40 µM), TNFα (40 ng/ml), RSL3 (1 µM), Torin1 (5 µM), Nec-1 (10 µM) and Fer-1 (5 µM). Error bars in all experiments indicate SEM; *P < 0.05 as determined by a Student´s t-test (unpaired, 2-tailed) or a 1-way ANOVA with a Dunnett’s post-hoc test.

To have a cellular system capable of undergoing multiple forms of cell death, we made use of the mammalian adherent L929 cell line derived from murine subcutaneous areolar and adipose tissue [29]. Firstly, we treated L929 cells with staurosporine, a phospholipid/calcium-dependent protein kinase inhibitor and common inducer of caspase dependent apoptosis [30] in L929 cells [31]. As expected, there was a robust induction of apoptosis as ascertained by Annexin V staining (Annexin V^+^/7AAD^-^ population, early apoptosis + Annexin V^+^/7AAD^+^ population, late apoptosis) 24 hours post-treatment (Fig. 1A). Next, when we treated L929 cells with the GPX4 inhibitor RSL3, a known inducer of ferroptotic cell death, we observed an increase in cell death which was entirely reversible by the addition of the ferroptosis inhibitor ferrostatin-1 (Fer-1) [32] (Fig. 1B). We corroborated the induction of ferroptosis and its reversibility in L929 cells by also measuring lipid ROS accumulation using BODIPY C11 staining (Fig. 1C) [32, 33]. Next, we treated L929 cells with TNF-α and the pan-caspase inhibitor zVAD to induce necroptosis. Indeed, we observed the induction of cell death which was reversed by the addition of Necrostatin-1 (Nec-1), a RIP1 kinase inhibitor and necroptosis blocker (Fig. 1D). Furthermore, we also induced autophagy using the mTORC1/2 inhibitor Torin1 and confirmed this through LC3 increases using immunofluorescence (Fig. 1E). In our study, we aimed to create a rapid system for cell death classification using morphological differences. Apoptosis leads to caspase-dependent cytoskeleton reorganization, followed by membrane blebbing and finally membrane vesicle generation, called apoptotic bodies [34, 35]. Characteristic apoptotic body accumulation was visible in L929 cells induced by staurosporine (Fig. 1F). A critical step during ferroptosis execution depends on lipid ROS accumulation that specifically leads to membrane lipid peroxidation [36]. Induction of ferroptosis results in damaged membrane integrity likely due to sterical causes within peroxidized polyunsaturated fatty acid (PUFA) tails and cell “ballooning” [37]. This was indeed observed upon RSL3 treatment (Fig. 1F). TNF-α-induced necroptosis through RIP kinase 1 and 3 interaction consequently leads to MLKL phosphorylation and its oligomerization, creating cell membrane pores [38]. Additionally, MLKL complex in cell membrane mediates recruitment of Na^+^ and Ca^2+^ ion channels [38]. Changes in cell membrane composition, but also cytoskeletal integration leads to cell membrane blebbing, without apoptotic body formation, and finally cell membrane rupture [39] (Fig. 1F). Although ferroptosis and necroptosis finally converge on the necrotic morphotype (Supplementary Fig. 1A) [1], the neural network will likely be able to discern distinguishing subtle and early occurring morphological changes. Autophagy, on the other hand, is a primarily cytoprotective process induced under stress or starvation conditions, but when prolonged and pharmacologically activated can also result in cell death [40]. Morphologically, autophagy is characterized by cytoplasmic accumulation of double membrane vesicles, autophagosomes, complexes that deliver cellular components to lysosome [40, 41]. In the case of autophagy induction upon the treatment with Torin 1, L929 cells became spindle-shaped and characterized by visible vacuole accumulation in cells (Fig. 1F).

### Retraining of a convolutional neural network using brightfield images predicts different models of cell death

**Fig. 2:**
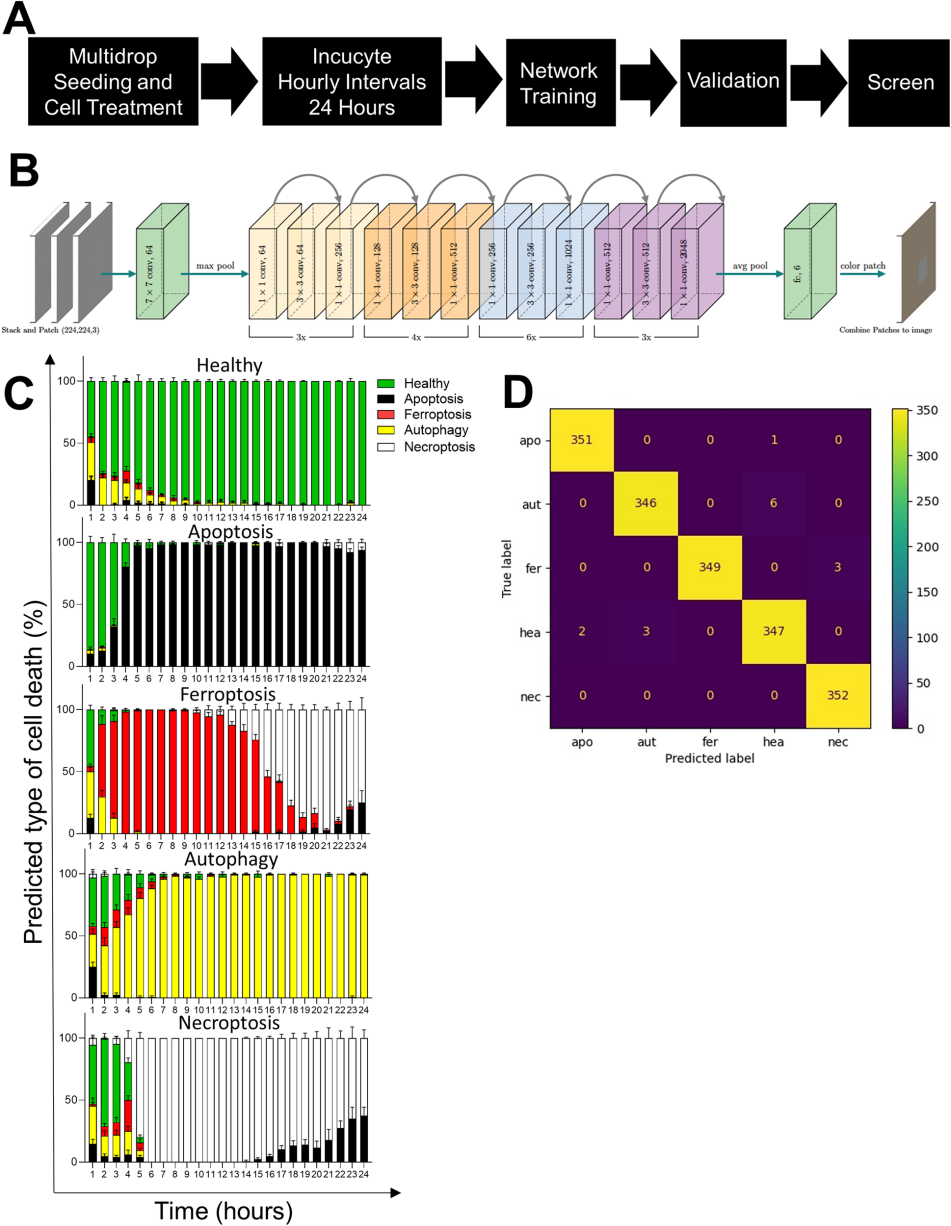
Deep transfer learning is used for automated recognition of different types of cell death. **A** Schematic representations of the workflow in training/fine-tunning CNN and drug screening and (**B**) CNN architecture and example of image classification are shown. **C** Deep learning image classification from L929 cells induced with the corresponding type of cell death stimuli during a 24 hour time course are shown. **D** Validation confusion matrix of cell death modes predicted by DTL ResNet50 versus final biological categorization is shown. The y-axis represents the evaluated labels and the x-axis the predicted labels of cell death. The color gradient indicates the number of images.

Next, we set up the Incucyte® Live-Cell Analysis imagining system microscope to acquire brightfield images of L929 cells taken every hour following treatment with the cell death inducers. One picture of each well of a 384 well plate was taken every hour for 24 hours (Fig. 2A) and this was repeated three independent times. In order to discern subtle morphological changes before any convergence on common morphotypes and before measurable cell death using biochemical, and/or other microscopic methods, the 8 hour time point was selected to train the Resnet50 neural network [22, 42]. Accordingly, to retrain ‘Resnet50′ to distinguish ferroptosis, apoptosis, necroptosis and autophagy from each other and healthy L929 cells, larger images (1408 ×1040) from the first two independent experiments were dissected into the required input images size (224 × 224) for ‘Resnet50′ using Adam as the optimizer (Fig. 2B). The third experiment was used for independent validation. In total, 108 images of each condition (healthy, apoptotic, ferroptotic, autophagic and necroptotic cells) were split into 64 images for training (dissected into 1408 images), 22 for validation and 22 for testing, each dissected into 352 images. The network could accurately predict all modes of cell death with almost 100% accuracy for the 8 hour time point (Fig. 2C, D), with color coded classification example in Supplementary Fig. 1B. An f1 score of 1.00 for apoptosis ferroptosis and necroptosis was achieved and 0.97 and 099 for autophagy and healthy cells respectively (Table 1). The network could predict the mode of cell death for the duration of the 24 hours for apoptosis and autophagy but at the later time points (past 14 hours) ferroptosis was classified as necroptosis which is expected as both necroptosis and ferroptosis eventually converge on the necrotic morphotype [1] (Supplementary Fig. 1A). Notably, at 1 hour post compound-treatment, cell death prediction was partially confounded by autophagy classifications, most likely attributable to cell perturbations when adding the cell death inducing compounds (Fig. 2C). Taken together, we have successfully applied a neural network to train bright field images to identify four different modes of cell death.

**Table 1:** Prediction accuracy of the trained nerural network.

Class	Precision	Recall	F1-score	Support
**Apoptosis**	1.00	1.00	1.00	176
**Autophagy**	0.99	0.97	0.98	176
**Ferroptosis**	1.00	1.00	1.00	176
**Healthy**	0.97	0.99	0.98	176
**Necroptosis**	1.00	1.00	1.00	176

### Retraining of a convolutional neural network can be applied to a high throughput screen

**Fig. 3:**
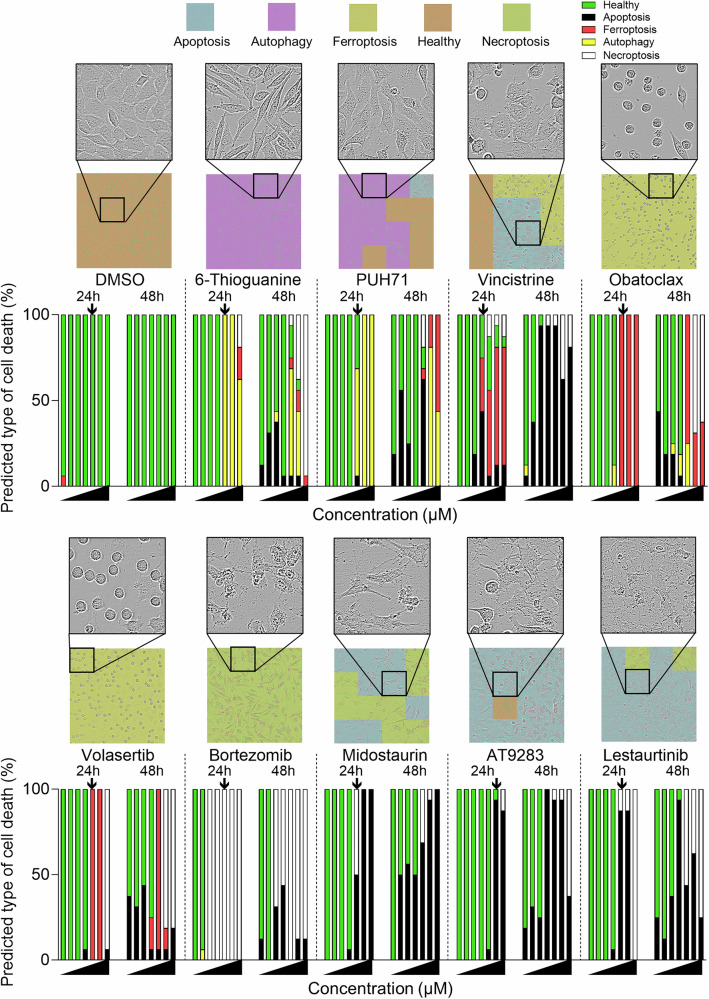
Deep transfer learning combined with drug screen successfully classifies 4 different modes of cell death. Brightfield images were acquired of L929 cells treated with increasing concentrations of drugs from a library of 84 compounds. Images were classified using the DLT learning program to identify different modes of cell death. Several examples of drugs and their classification at the 24 and 48-hour time-point are shown with the accompanying representative cell death classified image. The black arrow indicates the chosen concentration at which the magnified bright field image is shown.

L929 cells were screened using a library of clinically active chemotherapeutic and targeted-therapy anti-leukemia drugs at a wide concentration range (5 nM - 25 µM) [43]. As before, images were acquired every hour and fed into the convolutional neural network (CNN). As most of the drugs constituting the library have been in the clinic for years, their mode of action can be classically attributed to inducing apoptotic or anti-proliferative effects. As expected, cell death prediction by the CNN at the 24 and 48-hour time points at multiple drugs concentrations identified most drugs as being pro-apoptotic or having no effects in L929 cells (Table 2) with some exceptions. Bortezomib is clinically effective in the treatment of T-cell acute lymphoblastic leukemia and lymphoma [44] and has mainly been reported to induce apoptosis in leukemia and solid tumor cell lines [45]. Indeed, bortezomib-inducing modes cell death include immunogenic cell death [46], necroptosis as a single agent [47, 48] and in combination with toll-like receptors (TLRs). Strikingly, this capacity of bortezomib to also induce necroptosis under certain contexts was corroborated by our deep learning approach and screening strategy as evidenced by the color coded classification and zoomed in morphology (Fig. 3). In addition, the known autophagy inducer 6-Thioguanine [49] was successfully identified by our screening approach and interestingly, the HSP90 inhibitor PUH71 also induced autophagy in L929 cells. Moreover, known apoptosis inducing FLT3 inhibitors (midostaurin, lestaurib), AT9283 (Aurora Kinase) and others were correctly classified (Fig. 3 and Table 2) altogether verifying the validity of our novel screening approach. Vincristine suppresses microtubule assembly leading to cell cycle arrest and subsequently to autophagy, senescence or apoptosis, depending on the genetic background of the cancer cell [50]. In our system, vincristine was classified as inducing ferroptosis at the 24-hour time point and as inducing apoptosis at 48 hours. This example demonstrates the importance of considering multiple time-points and concentrations as well as thorough secondary validation analyses. Interestingly, several novel ferroptosis-inducing activities were identified amongst the molecules screened (Fig. 3 and Table 2). Notably, this includes volasertib, designed to function as a polo-like kinase (PLK) inhibitor with a high potency against mitosis-controlling PLK1, PLK2 and PLK3 [51]. Inhibition of PLK with volasertib has been shown to induce mitotic arrest, inhibit proliferation and induce apoptosis [51, 52]. The only report, to our knowledge, connecting volasertib with ferroptosis is a bioinformatics study in which primary breast cancer expression data with a ferroptosis-related gene signature was predicted to be responsive to volasertib treatment [53]. Therefore, we decided to further validate the ferroptosis-inducing functions of volasertib.

**Table 2:** Deep learning classification of induced cell death by a drug library.

Compound	Classified cell death	Compound class / target
Vinblastine	Ferroptosis	Antimitotics
Vincristine	Apoptosis	Antimitotics
Mitoxantrone	Necroptosis	Topoisomerase inhibitors
Daunorubicin	Necroptosis	Topoisomerase inhibitors
Amsacrine	Necroptosis/Apoptosis	Topoisomerase inhibitors
Dexamethasone	Apoptosis	GC/GCR complex
Prednisolone	Apoptosis	GC/GCR complex
Belinostat	Apoptosis	HDACi
CI-994	Apoptosis	HDACi
Panobinostat	Apoptosis	HDACi
Romidepsin	Apoptosis	HDACi
Entinostat	Apoptosis	HDACi
Ricolinostat	Apoptosis	HDAC6i
DMSO	Healthy	Control
5-Azacytidine	Apoptosis	Antimetabolites
6-Mercaptopurine	Autophagy	Antimetabolites
6-Thioguanine	Autophagy	Antimetabolites
Clofarabine	Apoptosis	Antimetabolites
Cytarabine	Apoptosis	Antimetabolites
Cyclocytidine HCL	Apoptosis	Antimetabolites
Nelarabine	Apoptosis	Antimetabolites
Methotrexate	Apoptosis	Antimetabolites
Aurora A Inhibitor I	Apoptosis	Aurora Kinase A inhibitor
Barasertib	Apoptosis	Aurora kinase B
Volasertib	Ferroptosis	Polo-like Kinase (PLK)
BI2536	Ferroptosis	PLK
LY2835219	Autophagy	CDK
Dinaciclib	Apoptosis	CDK
SY-1365-THZ1	Apoptosis	cdk7i
Axitinib	Apoptosis	VEGFR
Midostaurin	Apoptosis	PKC, vegfr2, pdgfr, FLT3
Dovitinib	Apoptosis	FLT3; PDGFR; VEGFR; c-Kit
Pexidartinib	Apoptosis	FLT3, KIT, CSF1R
Lestaurtinib	Apoptosis	FLT3
Quizartinib	Ferroptosis	FLT3
Decitabine	Healthy	Antimetabolites
DMSO	Healthy	Control
GSK343	Ferroptosis	EZH2i/HMTasei
Bortezomib	Necroptosis	Proteasome inhibitor
MLN-9708 (Citrate)	Necroptosis	Proteasome inhibitor
Ganetespib	Autophagy	HSP90i
PUH71	Autophagy	HSP90i
AUY922 (LUMINESPIB)	Autophagy	HSP90i
Alisertib	Apoptosis	Aurora Kinase A
CYT387	Apoptosis	JAK
Ruxolitinib	Apoptosis	JAK
Fedratinib	Apoptosis	JAK; FLT3
Tipifarnib	Apoptosis	Farnesyl Transferase; Ras inhibitor
Lonafarnib	Autophagy	Rasi
Sorafenib	Ferroptosis	Raf inhibitor
Regorafenib	Autophagy	Raf inhibitor
Cobimetinib	Apoptosis	MEK
MEK162	Apoptosis	MEK
Trametinib	Apoptosis	MEK
Tirabrutinib	Apoptosis	BTKi
Ibrutinib	Ferroptosis	(Ibruvica) ‘Btk
Imatinib	Apoptosis	Bcr-Abl inhibitor
DMSO	Healthy	Control
Pacritinib	Apoptosis	FLT3; JAK
Idelalisib	Healthy	PI3K
Dactolisib	Autophagy	PI3K, mTOR
Ro 08-2750	Apoptosis	P53mut
ARQ-092 Miransertib	Apoptosis	AKT
Everolimus	Autophagy	mTOR
Temsirolimus	Autophagy	mTOR
AT9283	Apoptosis	JAK
Obatoclax	Ferroptosis	Bcl-2 Family
QNZ	Autophagy	NF-κBi
Omaveloxolone	Ferroptosis	NF-κBi
Birinapant	Apoptosis	XIAPi and cIAP1i
Selinexor	Apoptosis	CRM1 inhibitor
AZD6738	Apoptosis	ATM/ATRi
Omacetaxine Mepes.	Apoptosis	Ribosom Inhibtor
Bexarotene	Apoptosis	Retinoid Inhibitor
Nintedanib	Apoptosis	LCK inhibitor
Copanlisib	Autophagy	PI3K
Palbociclib	Autophagy	CDKi
Birabresib	Apoptosis	Bromodomain (BRD2/3/4)
Tegaserod	Apoptosis	HTR4 agonist
5-nonyloxy tryptamine	Apoptosis	HTR1D agonist
DMSO	Healthy	Control
Ponatinib	Apoptosis	Bcr-Abl; FGFR; FLT3; VEGFR
Bosutinib	Apoptosis	Bcr-Abl; Src
Dasatinib	Autophagy	Bcr-Abl; Src
Staurosporin	Apoptosis	Non-selective inhibitor of protein kinases
BSI-201	Apoptosis	PARP
Olaparib	Apoptosis	PARP
Venetoclax	Apoptosis	Bcl-2 Family

### Subsets of precursor B-cell lymphoblastic leukemia (B-ALL) are sensitive to RSL3 and volasertib-mediated ferroptosis

**Fig. 4:**
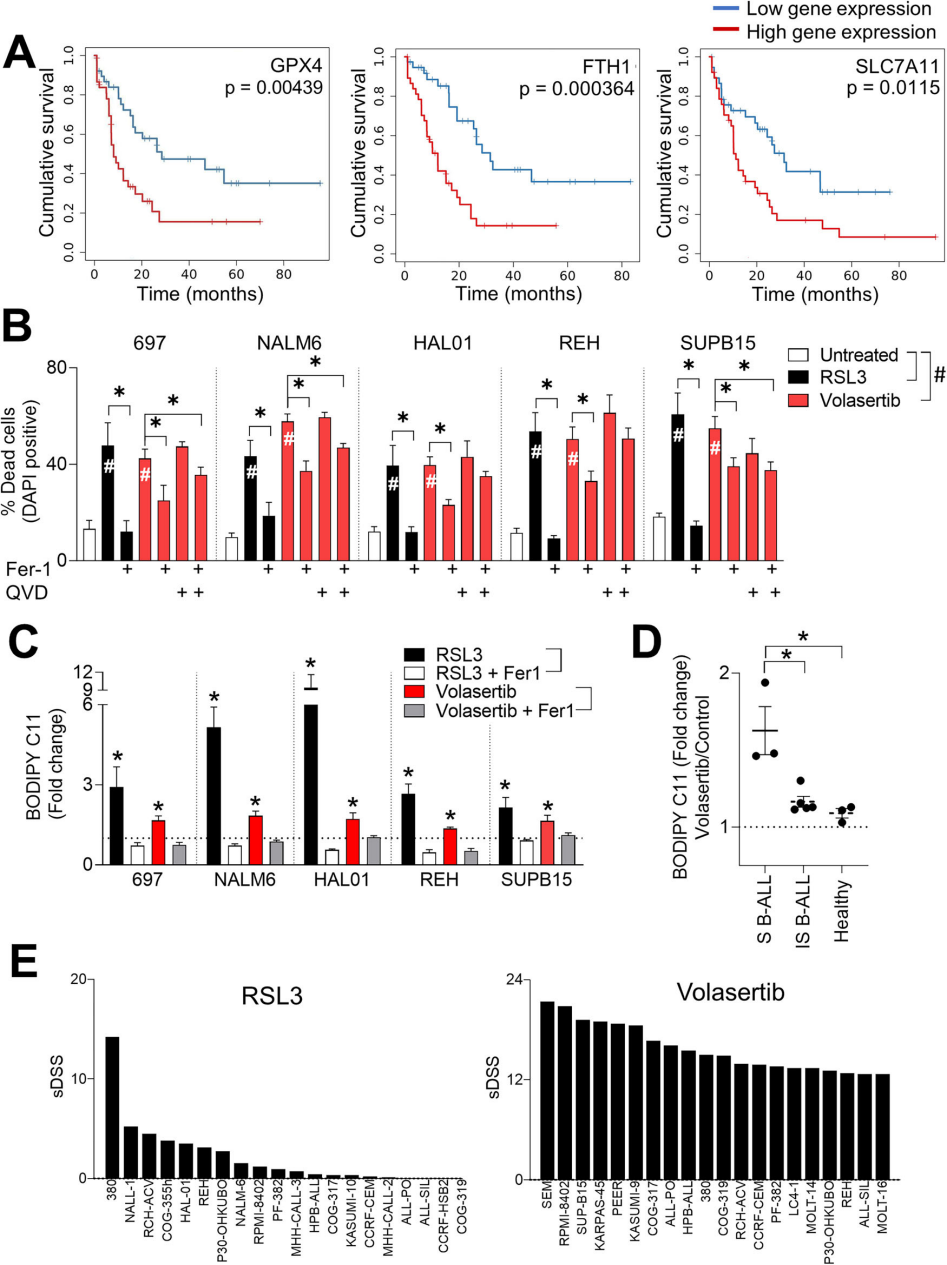
B-ALL cells are sensitive to ferroptosis induction. **A** Kaplan–Meier survival curves for patients with leukemia (*n* = 173, from the TCGA LAML cohort) were stratified according to *GPX4, FTH1* and *SLC7A11* transcript expression. **B** Cell death was measured by evaluating the percentage of DAPI positive cells using FACS after 72 hours treatment with 1 µM RSL3 or 6 µM volasertib, 5 µM Fer-1 and 10 µM QVD (*n* = 8-11). **C** Ferroptosis induction in several human B-ALL cell lines and (**D**) primary patient samples was assessed by measuring BODIPY C11 green fluorescent influx following treatment with RSL3 (1 µM) or volasertib (6 µM) alone and in combination with Fer-1 (5 µM) for 24 hours (*n* = 3-9). Primary patient samples were stratified as ferroptosis sensitive (S B-ALL) and ferroptosis insensitive (IS B-ALL). **E** Top 20 cell line selective drug sensitivity (sDSS) RSL3 and volasertib scores from the online FORALL resource are shown. Error bars in all experiments indicate SEM; *P < 0.05 as determined by a Student´s t-test (unpaired, 2-tailed) or a 1-way ANOVA with a Dunnett’s post-hoc test.

It has been suggested that cells that are resistant to other forms of cell death may be sensitive to ferroptosis [54]. Furthermore, the link between ferroptosis induction and sensitization to immunotherapies is an exciting approach to uncover novel tumor vulnerabilities [55, 56]. Notably, some hematological malignancies including leukemia have been suggested to be ferroptosis-sensitive in part due to an elevated steady-state oxidative stress potential [57, 58]. To understand if ferroptosis might impact leukemia patient survival, we mined data from the large gene expression collection of leukemia patient database (MILE study) [59] for patients’ survival stratified by expression of genes known to be involved in ferroptosis. Lower expression of major ferroptosis protective genes namely *GPX4, FTH1 and SLC7A11* correlated with significantly better survival (Fig. 4A). Although the functional consequences of this stratification are not clear in the context of actual ferroptosis induction, we reasoned that decreased *GPX4, FTH1 and SLC7A11* levels might facilitate ferroptosis induction in leukemia cells. We therefore firstly assessed whether cell death at 72 hours was induced in leukemia cells following treatment with a knownferroptosis inducer and GPX4 inhibitor RSL3 [60] in parallel to volasertib. We observed that some B-ALL subtypes namely 697 (TCF3::PBX1), NALM6 (DUX4-rearranged), SUPB15 (BCR::ABL1), HAL01 (TCF3::HLF) and the ETV6::RUNX1 positive REH’s had significantly reduced viability after RSL3 and volasertib treatment (Fig. 4B). Importantly, co-treatment with the synthetic anti-oxidant ferroptosis inhibitor, Fer-1 [12] was able to partially or fully rescue volasertib and RSL3 induced cell death respectively in B-ALL cell lines (Fig. 4B). Similar results were observed following co-treatment with the antioxidant vitamin E, α-tocopherol (α-TOC) (Supplementary Fig. 1C). When we additionally co-treated cells with volasertib and the PAN caspase inhibitor, QVD, neither QVD alone, or in combination with Fer-1 had significant rescue benefits in the context of volasertib-induced cell death. These rescue experiments indicate that while volasertib induced ferroptosis in B-ALL cell lines which led to cell death, there was also a contribution from other caspase-independent modes of cell death (Fig. 4B). To further validate whether volasertib and RSL3 induced ferroptosis, we quantified lipid ROS accumulation using the BODIPY C11 stain [33] 24 hours post-treatment. Both RSL3 and volasertib were able to significantly induce lipid peroxidation in several subtypes of B-ALL including 697, NALM6, HAL01, SUPB15 and REH. Importantly, lipid ROS accumulation was specifically reversed upon co-treatment with Fer-1 (Fig. 4C). Strikingly, treatment of NSG mice-transplanted primary patient B-ALL (primary B-ALL) but not healthy CD19^+^ cells purified from the blood of healthy donors resulted in significant BODIPY C11 accumulation following volasertib treatment (Fig. 4D).

Next, we wondered how the ability to induce ferroptosis translates into anti-cancer drug sensitivity in the context of a therapeutic index. We used a navigable drug sensitivity profile tool recently provided by the interactive online Functional Omics Resource of ALL (FORALL) [61] which includes both RSL3 and volasertib in its drug repertoire. FORALL assigns a selective drug sensitivity score (sDSS, using the CellTiter Glo cell viability assay) against 43 leukemia cells lines (25 BCP-ALL, 16 T-ALL, and 2 B-ALL) by normalizing to the response of normal bone marrow, a high sDSS thereby implying a high therapeutic index with low toxicity in healthy cells. The top twenty sDSS scores were plotted for each cell line (Fig. 4E). Only B-ALL cell lines has a discernible RSL3 sDSS score including the REH, HAL01 and NALM6 cells that we uncovered as being sensitive to ferroptosis induction using our deep-learning screening approach (Fig. 4E). Many hematological malignancies are exquisitely sensitive to the anti-proliferative/pro-apoptotic functions of volasertib [62–64] and this is reflected in the high sDSS scores for the drug across B/T-ALL cell lines. Sensitivity to volasertib does not necessarily correlate to ferroptosis induction and it is clear that volasertib selectively induces ferroptosis in specific B-ALL cell lines and primary leukemia patient samples. Additionally, we compared the kinetics of RSL3 and volasertib in inducing cell death directly overlayed with lipid peroxidation as measured using BODIPY C11. We observed that RSL3 had different kinetics than volasertib and induced cell death and ferroptosis at earlier time points (some at 8 hours post-treatment) in B-ALL cell lines (Supplementary Fig. 2).

### Volasertib induces a ferroptosis gene signature in B-ALL

**Fig. 5:**
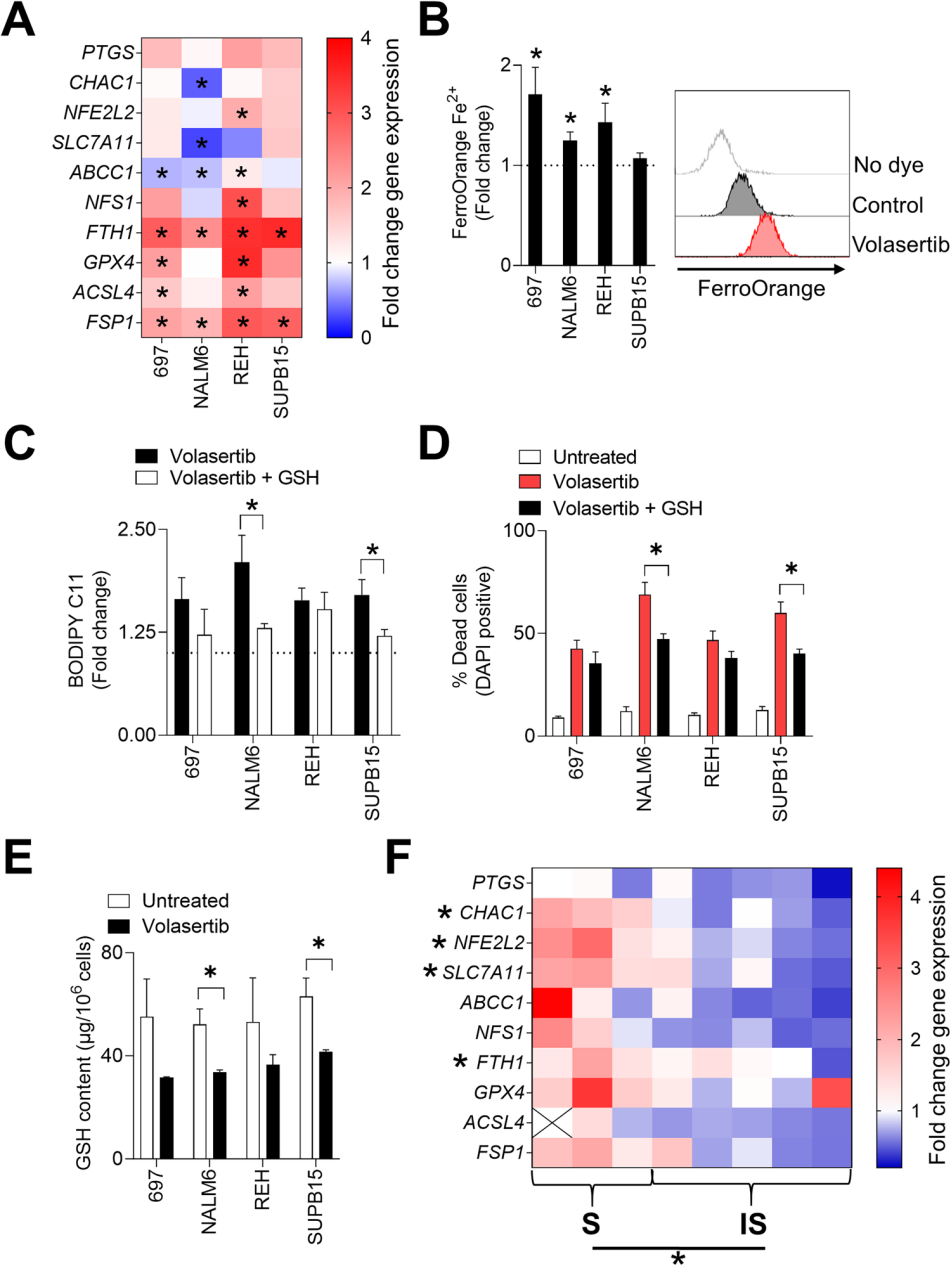
Sensitive B-ALL cells upregulate ferroptosis related genes post volasertib treatment. **A** Heat map representing fold changes of ferroptosis-related genes after 24 hours of treatment with 6 µM volasertib relative to untreated B-ALL cell lines is shown (*n* = 4-7). (**B**, left panel) B-ALL cells were treated with 6 µM volasertib for 24 hours and levels of Fe^2+^ were measured using FerroOrange dye by FACS. Relative levels compared to untreated cells are shown (*n* = 4-6). Representative shift of the signal is illustrated using the histogram on the right panel (representative histogram of n of 4-6 is shown). **C**, **D** B-ALL cells, treated with 6 µM volasertib and with supplementation of 5 mM GSH were FACS-analyzed after 24 hours for lipid peroxidation using BODIPY C11 in C (*n* = 3-5) or analyzed after 72 hours for DAPI positive cells in D (*n* = 4). **E** 10^6^ human B-ALL cells were treated with 6 µM volasertib for 24 hours and GSH was assessed from the cell pellet (*n* = 3). **F** Heat map representing fold changes of ferroptosis-related genes after 24 hours of treatment with 6 µM volasertib relative to untreated primary patient samples are shown (*n* = 8). S= ferroptosis sensitive. IS = ferroptosis insensitive. Error bars in all experiments indicate SEM; *P < 0.05 as determined by a Student´s t-test (unpaired, 2-tailed).

In contrast to other types of regulated cell death, the currently known molecular machinery necessary for ferroptosis hinges upon the collapse of the lipid ROS-specific antioxidant defenses. Mechanisms involved in ferroptosis are dependent on the interplay between the antioxidant pathway, control of cellular iron levels and metabolism of lipids [65, 66]. The antioxidant pathway depends on the cysteine transporter SLC7A11 and glutathione peroxidase 4 (GPX4) [67]. A main source of ROS during ferroptosis is through iron catalyzed in the Fenton reaction and to counteract this cells regulate levels of free iron by upregulating iron chelating ferritin (FTH1) [67, 68]. A distinct hallmark of ferroptosis is increased peroxidation of membrane phospholipids [69] where acyl-CoA synthetase long-chain family member 4 (ACSL4) plays a crucial role through the incorporation of polyunsaturated fatty acids (PUFAs) into membrane lipids [70]. Increased transcript levels of prostaglandin-endoperoxide synthase 2 (PTGS2) is also a characteristic ferroptosis marker [69]. We therefore analyzed transcriptional changes in cell lines and patient-derived B-ALL samples 24 hours post volasertib treatment. We observed an upregulation of antioxidant pathway genes NFE2L2, AIFM2, ACSL4 and GPX4 which was the most pronounced in the NALM6, 697 and REH’s (Fig. 5A). Similar to other studies [71], we also noticed increased mRNA levels of ferritin (FTH) suggesting an accumulation of iron. To test this, we stained cells with FerroOrange, a dye that labels ferrous (Fe^2+^) ions serving as a substrate for the ROS-producing Fenton reaction. Indeed, 24 hours post volasertib treatment, there was a robust accumulation of iron ions in B-ALL cell lines suggesting ferroptosis induction (Fig. 5B). Volasertib treatment downregulated expression of *SLC7A11* (Fig. 5A) suggesting lower levels of cellular glutathione (GSH), a main antioxidant and a substrate of GPX4, that can sensitize cells for ferroptosis [72]. When we supplemented volasertib treated cells with GSH, we observed a reversal of lipid peroxidation (Fig. 5C) and cell death (Fig. 5D) in NALM6 and SUPB15 cells. Additionally, when we analyzed GSH content of volasertib treated cells, we observed significantly lower glutathione in NALM6 and SUPB15 B-ALL cells (Fig. 5E). Interestingly, when we re-analyzed available online (GSE103068) RNA sequencing data of a volasertib-treated human AML cell line MV-4-11B [73], there was an activation of synthesis and metabolism of ROS (Supplementary Fig. 3A). Furthermore, confirming our results in B-ALL cells, when ferroptosis related genes were identified on a volcano plot from the publicly available AML RNA sequencing data, volasertib induced expression of NFE2L2, GPX4 and ABCC1 (Supplementary Fig. 3B). When we evaluated ferroptosis gene expression related changes in primary patient samples, the samples termed ferroptosis-sensitive according to their BODIPY C11 accumulation (Fig. 4D) responded to volasertib treatment by significantly upregulating CHAC1, NFE2L2, SLC7A11 and NFS1 (Fig. 5F). Taken together, we demonstrate that volasertib treatment induced the accumulation of ROS, iron ions and upregulated antioxidant pathway across leukemia cell lines and volasertib-responsive patient-derived B-ALL samples.

### Volasertib induces ferroptosis in vivo

**Fig. 6:**
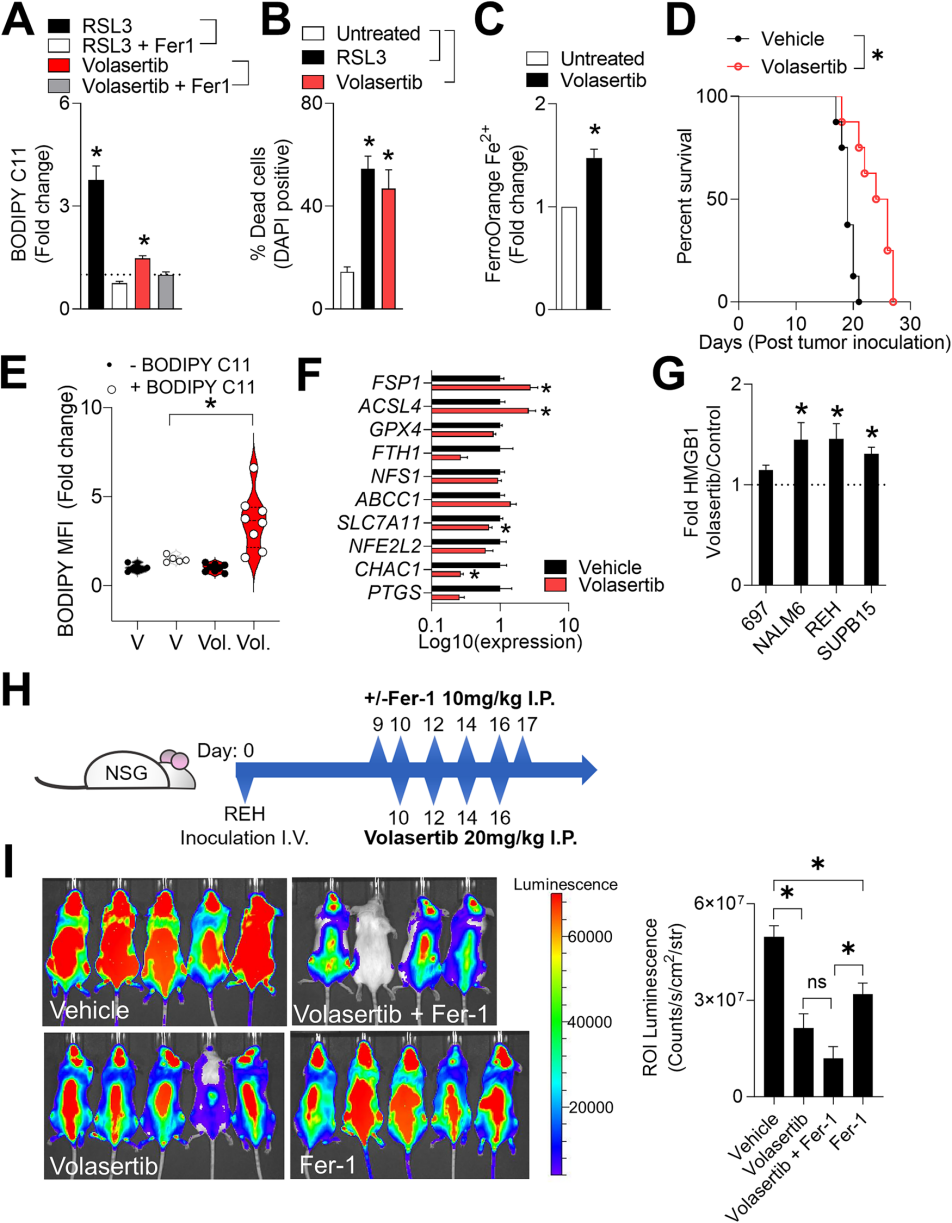
Volasertib induces ferroptosis and increases ACSL4 expression in vivo. **A** Lipid peroxidation was measured with BODIPY C11 in C1498 cells following treatment volasertib (6 µM) or RSL3 (1 µM) with or without Fer-1 (5 µM) for 24 hours (*n* = 5). **B** Viability of C1498 cells was measured by FACS and analyzed for the percentage of DAPI positive cells after 72 hours treatment with volasertib (6 µM) or RSL3 (1 µM, *n* = 8). **C** Level of Fe^2+^ in C1498 cells after 24 hours treatment with 6 µM volasertib was measured by staining with FerroOrange dye and assessed using FACS (*n* = 6). **D** C57BL/6 J mice were intravenously inoculated with 500,000 C1498-luc-GFP cells. After 7 days, mice were randomized by luminescent signal as assessed by IVIS and treated with 20 mg/kg volasertib or vehicle on day 7, 9, 11 and 13 post-inoculation. Survival was monitored (*n* = 8). **E**, **F** Schematic representation of the treatment and endpoint regimen is shown in Supplementary Fig. 4A. NSG mice were inoculated intravenously with 500,000 C1498-luc-GFP cells. Mice were randomized and treated with 20 mg/kg volasertib or vehicle on the indicated days. **E** Leukemia-engrafted spleens of volasertib or vehicle treated mice were stained with BODIPY C11 (*n* = 5-8 per group). **F** Expression of genes related to ferroptosis from leukemia-engrafted spleens of volasertib or vehicle treated mice is shown (*n* = 5-8). **G** Human B-ALL cells were treated with 6 µM volasertib and 48 hours later HMGB1 was measured from the supernatant using ELISA (*n* = 4). **H**, **I** NSG mice (*n* = 4-5 per group) were intravenously inoculated with 10^6^ REH-luc-GFP cells and after 6 days, randomized according to the IVIS bioluminescent imager into four treatment groups: vehicle, volasertib (20 mg/kg), Fer-1 (10 mg/kg) and volasertib + Fer-1. Mice were treated according to the schematic regimen in (**H**). 26 days post tumor inoculation, mice were IVIS scanned and engraftment signal was quantified in (**I**). Error bars in all experiments indicate SEM; *P < 0.05 as determined by a Student´s t-test (unpaired, 2-tailed) or a 1-way ANOVA with a Dunnett’s post-hoc test. For the Kaplan–Meier survival curve, the log-rank test was used.

Next, we wondered about the effects of volasertib in a syngeneic leukemia immuno-competent in vivo model. We utilized the C1498 murine leukemia cell line engineered to express GFP and luciferase (C1498-GFP-luc) [74, 75]. First, we confirmed ferroptosis induction by assessing ROS dependent lipid peroxidation using BODIPY C11. C1498 cells had a significantly increased level of BODIPY C11 upon RSL3 and volasertib treatment, which was reversed by pre-treatment with Fer-1 (Fig. 6A). Additionally, viability was decreased with RSL3 and volasertib treatment (Fig. 6B), suggesting induction of ferroptosis. Similar to human B-ALL we noticed iron accumulation in C1498 upon volasertib treatment (Fig. 6C). Next, we inoculated C57BL/6 J mice with C1498-GFP-luc, and, after 7 days, we randomized mice and treated them with 20 mg/kg volasertib or vehicle on day 7, 9, 11 and 13, as previously described [52, 62]. We observed prolonged survival of mice in the volasertib-treated group (Fig. 6D).

Next, we addressed whether volasertib induces similar changes in gene expression in vivo as observed in the ferroptosis-sensitive BCP-ALL cells in vitro. Previous studies have shown that T cell-derived interferon gamma (IFNγ) can promote ferroptosis in vivo [55]. To exclude any potential confounding effects of lymphoid immune infiltrating cells, we inoculated the immunocompromised NSG mice with C1498-GFP-luc cells. After 10 days, mice were IVIS scanned for tumor engraftment, randomized and treated with volasertib (Supplementary Fig. 4A, B). To further eliminate confounding effects of differing tumor sizes, we chose a time point where there were no differences in tumor burden (Supplementary Fig. 4B). We sacrificed mice and isolated GFP positive cells from engrafted organs, stained for 1 hour with BODIPY C11 and analyzed level of peroxided lipids. To account for constitutive GFP expression and baseline level of fluorescence, we included a BODIPY C11-unstained group. Only C1498-GFP-luc cells isolated from volasertib treated mice had elevated BODIPY C11, suggesting ongoing ferroptosis with increased level of ROS and membrane lipid peroxidation (Fig. 6E). Additionally, C1498-GFP-luc cells isolated from volasertib treated mice were characterized by increased AIFM2 and ACSL4 expression (Fig. 6F) in line with the ferroptosis gene signature observed in the in vitro system. Necroptosis, pyroptosis and ferroptosis represent a lytic type of cell death compared to non-lytic apoptosis. Lytic type of cell death can lead to release of intracellular matrix, together with immunogenic DAMPs including High Mobility Group Box 1 (HMGB1) [76]. We evaluated the release of HMGB1 by human cell lines 24 hours post volasertib treatment and found elevated HMGB1 levels in the supernatant (Fig. 6G). Additionally, we inoculated NSG mice with REH-luc-GFP and after randomization and checking for equal engraftment (Supplementary Fig. 4C), we treated the mice with vehicle, volasertib, Fer-1 and volasertib + Fer-1 (Fig. 6H). When tumor burden was analyzed 24 days post-inoculation using IVIS bioluminescent imaging, volasertib was able to decrease tumor burden compared to vehicle treated mice (Fig. 6I). Surprisingly, treatment with Fer-1 alone was also able to decrease tumor burden as well as having additive anti-cancer effects in combination with volasertib (Fig. 6I). When we also analyzed malondialdehyde (MDA, that accumulates as a result of lipid peroxidation) in the plasma on day 16, mice co-treated with Fer-1 had significantly lower levels of MDA suggesting homeostatic baseline ferroptosis in vehicle and volasertib treated mice (Supplementary Fig. 4D). Taken together, we demonstrated that volasertib induced ferroptosis in murine leukemic C1498 and human REH cells in vivo, in vitro and also prolonged survival in C1498 inoculated mice.

## Discussion

Artificial intelligence-based deep learning approaches have become indispensable in various applications used to for example classify organisms into specific taxa [77] or in medical imaging [78, 79]. In the case of pre-clinical research in the field of drug development, there is a need for rapid automated prediction of cell death, especially with the discovery of new modes of cell death. While others have used digital holographic microscopy to classify apoptosis and necroptosis [80], we created a pipeline that can be rapidly implemented, uses easily accessible brightfield images and can differentiate not only between apoptosis and necroptosis, but also autophagy and ferroptosis. Ferroptosis-induction is increasingly recognized as being a vulnerability in many types of cancers and its induction was recently demonstrated to circumvent drug resistance to apoptosis [65, 81]. Ferroptosis induction has been shown to be subtype and mutation specific [33, 82]. Studies have reported a connection between RAS mutations and sensitivity to ferroptosis induction, which led to the discovery of RSL3 (RAS-selective lethal 3) compound [60]. More recent literature supports a concept wherein RAS mutations render cells more resistant to ferroptosis via activation of NRF2 and ensuing FSP1 upregulation [83]. It was observed that cancers carrying the NRAS mutation differ in their Peroxisomal Biogenesis Factor 1 (PEX1) expression [84] that was reported to be crucial for generation of lipids with polyunsaturated acids making them sensitive to oxidation [85]. The 697, Nalm6 and Hal01 BCP-ALL cells also carry NRAS mutation and whether this property makes them sensitive to ferroptosis induction remains to be further explored. Others have shown that low levels of FSP1 also determine B-ALL sensitivity and upregulation of ferritin represents the major compensatory mechanism for ferroptosis-induction in leukemia [57]. Taken together, ferroptosis-induction is increasingly recognized as being a tumor vulnerability in hematological malignancies, particularly leukemia.

Interestingly, evidence of apoptosis induction following volasertib treatment has generally been limited to flow cytometric annexin V/PI cell assays which do not necessarily preclude ferroptosis induction [52, 73, 86, 87]. Thus, it is not clear if volasertib-induced apoptosis requires alternative cancer-type specific classifications as was recently demonstrated for sorafenib, which was found to induce ferroptosis and not apoptosis in some cancers [71, 88, 89]. Correct classification of cell death is especially important when it comes to immune activation, since ferroptosis, necroptosis and pyroptosis are considered immunogenic forms of cell death [90] known to induce “eat me” signals on their surface and release high levels of antigens and DAMPs [91]. In our system, volasertib treatment lead to release of HMGB1 in the supernatant in vitro and increased expression of ACSL4 in vitro and in vivo. This is particularly important as others have recently shown that elevated ACSL4 modulates cancer cell membrane lipid composition in vivo and makes them susceptible to T cell-derived IFNγ induced ferroptosis [55]. Taken together, ferroptosis induction has clinical implications especially in the context of immunotherapeutic combinatorial approaches [92].

Although RSL3 is very effective at inducing ferroptosis in vitro, its general toxicity [13] and poor pharmacokinetic precludes it from being utilized as a clinical compound. To potentially identify ferroptosis inducers which meet clinical criteria we specifically chose a clinically relevant small molecular library for the screen. PLK is overexpressed in many cancers and especially leukemia [93, 94], creating a therapeutic vulnerability. As such, volasertib entered phase II clinical trials for elderly patients with relapsed or refractory AML as a monotherapy or in combination with cytarabine resulting in increased complete response and prolonged survival [95, 96]. However, as this was not recapitulated in subsequent phase III clinical trials in chemotherapy-naive patients [97], it appears that responders are composed of relapsed or refractory patients as evidenced by currently open new phase II clinical trials designed at identifying further biomarkers of efficacy [98]. Notably, another PLK inhibitor used in our screen, BI2536 also induced ferroptosis (Table 2), suggesting connection between polo like kinases and the ferroptosis pathway. This should be further explored, taking in consideration recent study on cell cycle arrest sensitizing cancer cells to ferroptosis [99] and PLK properties in cell cycle.

In our study, we observed an upregulation of ferroptosis-related genes in B-ALL cell lines and the ferroptosis-sensitive primary B-ALL patient samples upon volasertib treatment. Several studies have also observed such changes [71, 100]. The upregulation of antioxidant pathway upon ferroptosis-induction may represent a compensatory mechanism that cells employ to counteract the effects of ferroptosis and this may have potential clinical implications in deriving a ferroptosis-responsive expression signature and identifying predictors of response sensitivity. It’s conceivable that cancer cells with dysregulated oxidative pathways are ferroptosis insensitive. Importantly, ferroptosis inducing agents are associated with overcoming chemoresistance as recent studies suggest that cancer cells resistant to typical apoptosis inducing molecules are highly dependent on GPX4 [54, 65], which could also potentially explain the successful outcome of volasertib in phase II of clinical trial on relapsed or refractory AML, but not on therapy-naive patients. Interestingly, we observed the highest MDA levels in the vehicle treated group (Supplementary Fig. 4D), potentially suggesting homeostatic spontaneous ferroptosis inhibited by Fer-1 treatment in the REH engrafted NSG mice resulting in decreased MDA levels. Surprisingly, in the context of tumor burden, treatment with Fer-1 had additive effects when combined with volasertib (Fig. 6I). Liproxstatin-1, another ferroptosis inhibitor was recently described as having anti-cancer effects through inhibiting spontaneous ferroptosis in neutrophils [101]. As NSG mice are reported to have functional myeloid immune cells including neutrophils, we propose that volasertib induces ferroptosis in engrafted cancer cells as well as immune cells, as neutrophils were reported to be sensitive to ferroptosis induction [102]. Fer-1 likely prevented cell death of neutrophils, leading to the additive therapeutic effect when combined with volasertib. However, this would have to be additionally evaluated in more detail and in the context of other immune populations in immune-competent mice.

## Material and methods

### Deep learning

For Comparison and Evaluation of our models we used the following five metrics, we show the metrics for the binary case.

The scores of the metrics are in the Interval [0, 1] and so greater the score so better.

$${Precision}=\frac{{TP}}{{TP}+{FP}}$$

$${Recall}=\frac{{TP}}{{TP}+{FN}}$$

$$F1-{Score}=\frac{2{TP}}{2{TP}+{FP}+{FN}}$$

$${Jaccard}-{Score}=\frac{{TP}}{{TP}+{FP}+{FN}}$$

$${Accuracy}=\frac{{TP}+{TN}}{{TP}+{FP}+{TN}+{FN}}$$

For the multiclass (not-binary) case the positive is the target class and the other classes are the negative class. With this definition, we get separate true positive (TP), false positive (FP), true negative (TN), false negative (FN) for each class and therefore separate metrics.

#### Classification score vector

We defined classification score vector that sums up the classification labels of each patch of an image and point the perceptual portion of this class from the image.

$$c:=\left({c}_{1},\ldots ,{c}_{i},\ldots ,{c}_{N}\right),$$

where i $\in$ 1, …, N and N is the number of classes.

With the definition

$$p:=\left({p}_{1},\ldots ,{p}_{j},\ldots ,{p}_{M}\right),$$

where j $\in$ $1,\ldots ,{\rm{M}}$ and M is the number of patches for this image.

Then we defined

$${c}_{i}:=\frac{{\sum }_{i=1}^{M}{1}_{f\left({p}_{i}={c}_{i}\right\}}}{{\sum }_{i=1}^{M}1},$$

where f is the prediction function of the neural network. The dominator guarantees that the sum of the vector entries is equal to one.

##### Architecture

We used a deep transfer learning approach for our architecture [103] and chose to fine-tune (num_train_layers = 3) the convolutional neural network Resnet50 [42] and adapted previously published code [28] for our experiments. The program generated for this project is available at https://github.com/immunooncol/pycedei.git. To fit the single color channel images (224,224,1) into the input format (three color channel (224,224,3)) of the ResNet50, we stack the same image three times. We used a square image patch size of 224 pixels.

##### Training

We trained the network with the batch size of 100 and 50 epochs, Early Stopping of 25 on the images of the samples from the data set (n_train_ = 1024 patches for each class) using the expert annotations data set as ground truth. The predicted probability for each image patch to contain each of the labels (‘apo’, ‘aut’, ‘fer’, ‘hea’, ‘nec’) was used as the objective/loss function (Cross Entropy Loss) in the training. We used Adam as the optimizer for this deep transfer learning approach and initial learning rate of 0.0001 and a decrease it by 5% each five epochs. Training and validation was performed on a Nvidia A100 of the high performance cluster (HPC, Hilbert) of the HHU, and on Quadro T2000 with Max-Q Design (Nvidia Corp., Santa Clara, CA, USA), depending on the computational power needed.

##### Evaluation

Evaluation was carried out by applying the previously trained model to the remaining, previously unseen data set (n_val_ = 352, n_test_ = 352 patches for each class) for each sequence set separately and comparing the results with the expert annotations as supplied by the biologist. In addition to the accuracy, we calculated the confusion matrix, the precision, recall, Jaccard index and the F1-score for each class. For Visualization we color each image patch in the color of the predicted class.

#### Software

We used the Python VERSION:3.8.8 [MSC v.1916 64 bit (AMD64)] software (pyTorch VERSION:1.9.0.dev20210423, CUDNN VERSION:8005). On the high-performance cluster we used the following software: Python VERSION:3.6.5 [GCC Intel(R)\\ C + + gcc 4.8.5 mode] (including pyTorch VERSION:1.8.0.dev20201102+cu110, CUDNN VERSION:8004).

### Cell culture

697 (*TCF3::PBX1* + ), REH (*ETV6::RUNX1* + ), NALM6 (*DUX4*-rearanged) and HAL01 (*TCF3::HLF* + ) cells were maintained in RPMI 1640 medium supplemented with 10% fetal calf serum (FCS) and glutamine. SUPB15 (*BCR::ABL1* + ) cells were cultivated in McCoy’s modified medium supplemented with 20% FCS. L929, C1498 (ATCC) and modified C1498-luc-GFP (stably expressing luciferase and GFP) were maintained in Dulbecco’s Modified Eagle’s medium with 10% FCS. All media were additionally supplemented with penicillin and streptomycin. Cells were incubated at 37 ^o^C in 5% CO2. Human B-ALL cell lines were purchased from (DSMZ) and were additionally identified using short tandem repeats (STR) profiling. Cell cultures were regularly controlled for Mycoplasma negativity using the MycoAlert Detection Kit (Lonza). Staurosporine, Torin1, Nec-1, zVAD, RSL3, Fer-1, α-TOC, GSH and volasertib (S2235) were purchased from Selleckchem and dissolved in DMSO or GSH in PBS. Recombinant murine TNF-α was purchased from R&D Systems and dissolved in PBS.

### Flow cytometry

Cells were washed twice with FACS buffer (PBS with 1% FCS and 5 mM EDTA) or Annexin buffer (88-8102-72 ThermoFisher) followed by Annexin V staining. Next, cells were incubated with 7AAD or DAPI and analyzed using FACS (CytoFLEX, BeckmanCoulter). For lipid peroxidation measurements, cells were additionally incubated for the last hour of treatment with 2.5 µM BODIPY C11 (D3861, ThermoFisher). FerroOrange (#36104, Cell Signaling) staining was performed according to the manufacturer’s instructions. Experiments were analyzed using FlowJo software. Graphs indicate an MFI fold change of treated / untreated for BODIPY C11 and FerroOrange.

### Drug screening and incucyte measurements

L929 cells were seeded on a flat bottom 96-well plate using Multidrop (Thermo Fisher). The next day cells were treated with the indicated compounds and incubated in the Incucyte machine (Sartorius). Leukemia-relevant drugs were dispensed onto a 384-well plate using the Digital Dispenser D300e (Tecan) in randomized well positions for drug screening [104]. Next, L929 cells were seeded using Multidrop and incubated in the Incucyte. Brightfield pictures using the Incucyte were taken at an interval of one hour for 24 hours.

### Real-time qPCR

Cells were lysed with QIAzol Lysis Reagent (QIAGEN) and RNA was isolated according to the manufacturer’s instructions. RNA was transcribed to cDNA (GoScript, Promega) and real-time qPCR analysis was performed according to manufacturer’s instructions (GoTaq, Promega).

### GSH, HMGB1 and MDA measurements

Equal amounts of human cells were seeded in a 24-well plate and treated with 6 µM volasertib for 24 hours for GSH and MDA or 48 hours for HMGB1 measurement. Next, the supernatant was collected and human HMGB1 ELISA (Invitrogen EEL047) was performed according to the manufacturer instructions. Cell pellets were sonicated with Ultrasonic Processor (UP100H, Hielscher) and GSH was measured using a Colorimetric Assay Kit (Elabscience, E-BC-K030-M) according to the manufacturer instructions. The malondialdehyde (MDA) colorimetric assay was performed from murine plasma (EEA015, Invitrogen) according to the manufacturer instructions.

### Mice and in vivo treatments

Wildtype C57BL/6 J and immunocompromised NSG (NOD.Cg-Prkdc^scid^IL2rg^tm1Wjl^/SzJ, JAX stock #005557) mice were maintained in pathogen-free conditions and for the duration of the experiment transferred to standard-barrier conditions. Mice were injected intravenously with 5 × 105 C1498-luc-GFP or 10^6^ REH-luc-GFP cells. IVIS scans were performed on the indicated days, 10 minutes after intraperitoneal injection of 10 µl/g of D-Luciferin (at a concentration of 15 mg/ml). Scans were performed with the IVIS In Vivo Imaging System (Perkin Elmer) and luminescence signal was analyzed. On day 10, mice were randomized according to engraftment signal (no blinding was performed) and treated with vehicle, volasertib (Selleckchem, S2235, 20 mg/kg) and Fer-1 (Selleckchem, S7243, 10 mg/kg) on the indicated days. Experiments were performed under the authorization of LANUV in accordance with the German law for animal protection.

### Patient derived cells

Primary patient leukemia blasts were obtained from BioBank, University Clinic Duesseldorf, after obtaining informed consent in accordance with the Declaration of Helsinki. The experiments were approved by the ethics committee of the Heinrich Heine University medical faculty (Study Nr.: 2019-566). The patient cells were intravenously passaged in NSG mice [43]. Engraftment of the leukemia cells was verified regularly through examination of the human CD45^+^ population in peripheral blood four weeks post-inoculation by flow cytometry. Mice were sacrificed at predefined endpoints, where more than 90% of human CD45^+^ cells were isolated from the BM and spleen of the mice with mouse cell depletion kit (Miltenyi Biotec). The isolated cells were short-term cultivated in RPMI 1640 Glutamax medium (Gibco) supplemented with 15% FCS, 1 mM Sodium Pyruvate (Gibco), 0.1 mM 2-Mercaptoethanol (Gibco) and 0.5 µg/ml Gentamicin (Invitrogen).

### Data mining

Kaplan–Meier survival curve data was generated with TIMER2.0 database. Expression of GPX4, SLC7A11 and FHC1 in patients and healthy bone marrow samples were extracted from Microarray Innovations in Leukemia (MILE) database. RNA-sequencing data was taken from open-source NCBI Gene Expression Omnibus (GEO) with accession number GSE103068. Gene expression data were analyzed in-house with Partek flow default settings.

### Statistics

The experiments were reproduced with a minimum of three biological replicates, precise number of replicates is indicated in the figure legend for each experiment. Error bars in each experiment indicate standard error of mean (SEM). Statistical analyses were performed using Prism v8.0.2 (GraphPad Software, La Jolla, CA, USA) or using Partek for RNA sequencing. Statistical test used is indicated in the Figure legends under each experiment. Statistical significance is indicated as *p values < 0.05.

## Supplementary information

supplementary figures

## Data Availability

The program generated for this project is available at https://github.com/immunooncol/pycedei.git. RNA-sequencing data was taken from available NCBI GEO with the accession ID: GSE103068.

## References

[1] Galluzzi L, Vitale I, Aaronson SA, Abrams JM, Adam D, Agostinis P, et al. Molecular mechanisms of cell death: recommendations of the Nomenclature Committee on Cell Death 2018. Cell Death Differ. 2018;25:486–541. 29362479 10.1038/s41418-017-0012-4 PMC5864239

[2] Zheng TS, Schlosser SF, Dao T, Hingorani R, Crispe IN, Boyer JL, et al. Caspase-3 controls both cytoplasmic and nuclear events associated with Fas-mediated apoptosis in vivo. Proc Natl Acad Sci USA. 1998;95:13618–23. 9811849 10.1073/pnas.95.23.13618 PMC24868

[3] Inoue S, Browne G, Melino G, Cohen GM. Ordering of caspases in cells undergoing apoptosis by the intrinsic pathway. Cell Death Differ. 2009;16:1053–61. 19325570 10.1038/cdd.2009.29

[4] Walsh JG, Cullen SP, Sheridan C, Luthi AU, Gerner C, Martin SJ. Executioner caspase-3 and caspase-7 are functionally distinct proteases. Proc Natl Acad Sci USA. 2008;105:12815–9. 18723680 10.1073/pnas.0707715105 PMC2529079

[5] Vitale I, Pietrocola F, Guilbaud E, Aaronson SA, Abrams JM, Adam D, et al. Apoptotic cell death in disease-Current understanding of the NCCD 2023. Cell Death Differ. 2023;30:1097–154. 37100955 10.1038/s41418-023-01153-w PMC10130819

[6] Delbridge AR, Grabow S, Strasser A, Vaux DL. Thirty years of BCL-2: translating cell death discoveries into novel cancer therapies. Nat Rev Cancer. 2016;16:99–109. 26822577 10.1038/nrc.2015.17

[7] Dickens LS, Boyd RS, Jukes-Jones R, Hughes MA, Robinson GL, Fairall L, et al. A death effector domain chain DISC model reveals a crucial role for caspase-8 chain assembly in mediating apoptotic cell death. Mol Cell. 2012;47:291–305. 22683266 10.1016/j.molcel.2012.05.004 PMC3477315

[8] Muzio M, Chinnaiyan AM, Kischkel FC, O’Rourke K, Shevchenko A, Ni J, et al. FLICE, a novel FADD-homologous ICE/CED-3-like protease, is recruited to the CD95 (Fas/APO-1) death–inducing signaling complex. Cell. 1996;85:817–27. 8681377 10.1016/s0092-8674(00)81266-0

[9] Seidel E, von Karstedt S. Extrinsic cell death pathway plasticity: a driver of clonal evolution in cancer?. Cell Death Discov. 2022;8:465. 36435845 10.1038/s41420-022-01251-7 PMC9701215

[10] Linkermann A, Green DR. Necroptosis. N Engl J Med. 2014;370:455–65. 24476434 10.1056/NEJMra1310050 PMC4035222

[11] Murphy JM, Czabotar PE, Hildebrand JM, Lucet IS, Zhang JG, Alvarez-Diaz S, et al. The pseudokinase MLKL mediates necroptosis via a molecular switch mechanism. Immunity. 2013;39:443–53. 24012422 10.1016/j.immuni.2013.06.018

[12] Dixon SJ, Lemberg KM, Lamprecht MR, Skouta R, Zaitsev EM, Gleason CE, et al. Ferroptosis: an iron-dependent form of nonapoptotic cell death. Cell. 2012;149:1060–72. 22632970 10.1016/j.cell.2012.03.042 PMC3367386

[13] Yang WS, SriRamaratnam R, Welsch ME, Shimada K, Skouta R, Viswanathan VS, et al. Regulation of ferroptotic cancer cell death by GPX4. Cell. 2014;156:317–31. 24439385 10.1016/j.cell.2013.12.010 PMC4076414

[14] Klionsky DJ, Petroni G, Amaravadi RK, Baehrecke EH, Ballabio A, Boya P, et al. Autophagy in major human diseases. EMBO J. 2021;40:e108863. 34459017 10.15252/embj.2021108863 PMC8488577

[15] Galluzzi L, Aaronson SA, Abrams J, Alnemri ES, Andrews DW, Baehrecke EH, et al. Guidelines for the use and interpretation of assays for monitoring cell death in higher eukaryotes. Cell Death Differ. 2009;16:1093–107. 19373242 10.1038/cdd.2009.44 PMC2757140

[16] Kepp O, Galluzzi L, Lipinski M, Yuan J, Kroemer G. Cell death assays for drug discovery. Nat Rev Drug Discov. 2011;10:221–37. 21358741 10.1038/nrd3373

[17] Havaei M, Davy A, Warde-Farley D, Biard A, Courville A, Bengio Y, et al. Brain tumor segmentation with deep neural networks. Medical image Anal. 2017;35:18–31. 10.1016/j.media.2016.05.004 27310171

[18] Kronberg RM, Meskelevicius D, Sabel M, Kollmann M, Rubbert C, Fischer I. Optimal acquisition sequence for AI-assisted brain tumor segmentation under the constraint of largest information gain per additional MRI sequence. Neuroscience Inform. 2022;2:100053.

[19] Jaiswal AK, Tiwari P, Kumar S, Gupta D, Khanna A, Rodrigues JJ. Identifying pneumonia in chest X-rays: a deep learning approach. Measurement. 2019;145:511–8.

[20] Huang L, Han R, Ai T, Yu P, Kang H, Tao Q, et al. Serial quantitative chest CT assessment of COVID-19: a deep learning approach. Radiology: Cardiothorac Imaging. 2020;2:e200075. 10.1148/ryct.2020200075 PMC7233442 33778562

[21] Pan J, Hong G, Zeng H, Liao C, Li H, Yao Y, et al. An artificial intelligence model for the pathological diagnosis of invasion depth and histologic grade in bladder cancer. J Transl Med. 2023;21:42. 36691055 10.1186/s12967-023-03888-z PMC9869632

[22] Kronberg RM, Haeberle L, Pfaus M, Xu HC, Krings KS, Schlensog M, et al. Communicator-Driven Data Preprocessing Improves Deep Transfer Learning of Histopathological Prediction of Pancreatic Ductal Adenocarcinoma. Cancers (Basel). 2022;14:1964. 35454869 10.3390/cancers14081964 PMC9031738

[23] Sun T, Wang Y, Liu X, Li Z, Zhang J, Lu J, et al. Deep learning based on preoperative MR images improves the predictive power of survival models in primary spinal cord astrocytomas. Neuro Oncol. 2022;25:1157–65. 10.1093/neuonc/noac280 PMC10237430 36562243

[24] Shamai G, Livne A, Polonia A, Sabo E, Cretu A, Bar-Sela G, et al. Deep learning-based image analysis predicts PD-L1 status from H&E-stained histopathology images in breast cancer. Nat Commun. 2022;13:6753. 36347854 10.1038/s41467-022-34275-9 PMC9643479

[25] Esteva A, Kuprel B, Novoa RA, Ko J, Swetter SM, Blau HM, et al. Dermatologist-level classification of skin cancer with deep neural networks. Nature. 2017;542:115–8. 28117445 10.1038/nature21056 PMC8382232

[26] La Greca AD, Perez N, Castaneda S, Milone PM, Scarafia MA, Mobbs AM, et al. celldeath: A tool for detection of cell death in transmitted light microscopy images by deep learning-based visual recognition. PLoS One. 2021;16:e0253666. 34166446 10.1371/journal.pone.0253666 PMC8224851

[27] Linsley JW, Linsley DA, Lamstein J, Ryan G, Shah K, Castello NA, et al. Superhuman cell death detection with biomarker-optimized neural networks. Sci Adv. 2021;7:eabf8142. 34878844 10.1126/sciadv.abf8142 PMC8654296

[28] Werner J, Kronberg RM, Stachura P, Ostermann PN, Muller L, Schaal H, et al. Deep transfer learning approach for automatic recognition of drug toxicity and inhibition of SARS-CoV-2. Viruses. 2021;13:610. 33918368 10.3390/v13040610 PMC8066066

[29] Sanford KK, Earle WR, Likely GD. The growth in vitro of single isolated tissue cells. J Natl Cancer Inst. 1948;9:229–46. 18105872

[30] Belmokhtar CA, Hillion J, Segal-Bendirdjian E. Staurosporine induces apoptosis through both caspase-dependent and caspase-independent mechanisms. Oncogene. 2001;20:3354–62. 11423986 10.1038/sj.onc.1204436

[31] Humphreys DT, Wilson MR. Modes of L929 cell death induced by TNF-alpha and other cytotoxic agents. Cytokine. 1999;11:773–82. 10525316 10.1006/cyto.1998.0492

[32] Alborzinia H, Florez AF, Kreth S, Bruckner LM, Yildiz U, Gartlgruber M, et al. MYCN mediates cysteine addiction and sensitizes neuroblastoma to ferroptosis. Nat Cancer. 2022;3:471–85. 35484422 10.1038/s43018-022-00355-4 PMC9050595

[33] Bebber CM, Thomas ES, Stroh J, Chen Z, Androulidaki A, Schmitt A, et al. Ferroptosis response segregates small cell lung cancer (SCLC) neuroendocrine subtypes. Nat Commun. 2021;12:2048. 33824345 10.1038/s41467-021-22336-4 PMC8024350

[34] Battistelli M, Falcieri E. Apoptotic bodies: particular extracellular vesicles involved in intercellular communication. Biology. 2020;9:21. 31968627 10.3390/biology9010021 PMC7168913

[35] Oropesa-Ávila M, Fernández-Vega A, de la Mata M, Maraver JG, Cordero MD, Cotán D, et al. Apoptotic microtubules delimit an active caspase free area in the cellular cortex during the execution phase of apoptosis. Cell Death Dis. 2013;4:e527. 23470534 10.1038/cddis.2013.58 PMC3613836

[36] Agmon E, Solon J, Bassereau P, Stockwell BR. Modeling the effects of lipid peroxidation during ferroptosis on membrane properties. Scientific Rep. 2018;8:5155. 10.1038/s41598-018-23408-0 PMC5979948 29581451

[37] Dodson M, Castro-Portuguez R, Zhang DD. NRF2 plays a critical role in mitigating lipid peroxidation and ferroptosis. Redox Biol. 2019;23:101107. 30692038 10.1016/j.redox.2019.101107 PMC6859567

[38] Dhuriya YK, Sharma D. Necroptosis: a regulated inflammatory mode of cell death. J Neuroinflammation. 2018;15:199. 29980212 10.1186/s12974-018-1235-0 PMC6035417

[39] Shkarina K, Hasel de Carvalho E, Santos JC, Ramos S, Leptin M, Broz P. Optogenetic activators of apoptosis, necroptosis, and pyroptosis. J Cell Biol. 2022;221:e202109038. 35420640 10.1083/jcb.202109038 PMC9014795

[40] Wirawan E, Vanden Berghe T, Lippens S, Agostinis P, Vandenabeele P. Autophagy: for better or for worse. Cell Res. 2012;22:43–61. 21912435 10.1038/cr.2011.152 PMC3351915

[41] Parzych KR, Klionsky DJ. An overview of autophagy: morphology, mechanism, and regulation. Antioxidants Redox Signal. 2014;20:460–73. 10.1089/ars.2013.5371 PMC3894687 23725295

[42] He K, Zhang X, Ren S, Sun J, editors. Deep residual learning for image recognition. Proceedings of the IEEE conference on computer vision and pattern recognition; 2016.

[43] Vogt M, Dienstbier N, Schliehe-Diecks J, Scharov K, Tu JW, Gebing P, et al. Co-targeting HSP90 alpha and CDK7 overcomes resistance against HSP90 inhibitors in BCR-ABL1+ leukemia cells. Cell Death Dis. 2023;14:799. 38057328 10.1038/s41419-023-06337-3 PMC10700369

[44] Teachey DT, Devidas M, Wood BL, Chen Z, Hayashi RJ, Hermiston ML, et al. Children’s Oncology Group Trial AALL1231: A Phase III Clinical Trial Testing Bortezomib in Newly Diagnosed T-Cell Acute Lymphoblastic Leukemia and Lymphoma. J Clin Oncol. 2022;40:2106–18. 35271306 10.1200/JCO.21.02678 PMC9242409

[45] Chu YY, Ko CY, Wang SM, Lin PI, Wang HY, Lin WC, et al. Bortezomib-induced miRNAs direct epigenetic silencing of locus genes and trigger apoptosis in leukemia. Cell Death Dis. 2017;8:e3167. 29120412 10.1038/cddis.2017.520 PMC5775404

[46] Gulla A, Morelli E, Samur MK, Botta C, Hideshima T, Bianchi G, et al. Bortezomib induces anti-multiple myeloma immune response mediated by cGAS/STING pathway activation. Blood Cancer Discov. 2021;2:468–83. 34568832 10.1158/2643-3230.BCD-21-0047 PMC8462183

[47] Wang YC, Wang LT, Hung TI, Hong YR, Chen CH, Ho CJ, et al. Severe cellular stress drives apoptosis through a dual control mechanism independently of p53. Cell Death Discov. 2022;8:282. 35680784 10.1038/s41420-022-01078-2 PMC9184497

[48] Moriwaki K, Chan FK. Regulation of RIPK3- and RHIM-dependent necroptosis by the proteasome. Journal Biol Chem. 2016;291:5948–59. 26786097 10.1074/jbc.M115.700997 PMC4786728

[49] Zeng X, Kinsella TJ. Mammalian target of rapamycin and S6 kinase 1 positively regulate 6-thioguanine-induced autophagy. Cancer Res. 2008;68:2384–90. 18381446 10.1158/0008-5472.CAN-07-6163

[50] Filippi-Chiela EC, Vargas JE, Bueno E, Silva MM, Thomé MP, Lenz G. Vincristine promotes differential levels of apoptosis, mitotic catastrophe, and senescence depending on the genetic background of glioblastoma cells. Toxicology Vitr. 2022;85:105472. 10.1016/j.tiv.2022.105472 36116745

[51] Rudolph D, Steegmaier M, Hoffmann M, Grauert M, Baum A, Quant J, et al. BI 6727, a Polo-like kinase inhibitor with improved pharmacokinetic profile and broad antitumor activity. Clinical cancer research : an official journal of the. American Assoc Cancer Res. 2009;15:3094–102. 10.1158/1078-0432.CCR-08-2445 19383823

[52] Gjertsen BT, Schöffski P. Discovery and development of the Polo-like kinase inhibitor volasertib in cancer therapy. Leukemia. 2015;29:11–9. 25027517 10.1038/leu.2014.222 PMC4335352

[53] Peng Y, Yu H, Zhang Y, Qu F, Tang Z, Qu C, et al. A ferroptosis-associated gene signature for the prediction of prognosis and therapeutic response in luminal-type breast carcinoma. Scientific Rep. 2021;11:17610. 10.1038/s41598-021-97102-z PMC8413464 34475496

[54] Hangauer MJ, Viswanathan VS, Ryan MJ, Bole D, Eaton JK, Matov A, et al. Drug-tolerant persister cancer cells are vulnerable to GPX4 inhibition. Nature. 2017;551:247–50. 29088702 10.1038/nature24297 PMC5933935

[55] Liao P, Wang W, Wang W, Kryczek I, Li X, Bian Y, et al. CD8(+) T cells and fatty acids orchestrate tumor ferroptosis and immunity via ACSL4. Cancer Cell. 2022;40:365–78.e6. 35216678 10.1016/j.ccell.2022.02.003 PMC9007863

[56] Gong D, Chen M, Wang Y, Shi J, Hou Y. Role of ferroptosis on tumor progression and immunotherapy. Cell Death Discov. 2022;8:427. 36289191 10.1038/s41420-022-01218-8 PMC9605952

[57] Lalonde ME, Sasseville M, Gelinas AM, Milanese JS, Beland K, Drouin S, et al. Genome-wide CRISPR screens identify ferroptosis as a novel therapeutic vulnerability in acute lymphoblastic leukemia. Haematologica. 2022;108:382–393. 10.3324/haematol.2022.280786 PMC9890019 36134452

[58] Zhao Y, Huang Z, Peng H. Molecular mechanisms of ferroptosis and its roles in hematologic malignancies. Front Oncol. 2021;11:743006. 34778060 10.3389/fonc.2021.743006 PMC8582018

[59] Haferlach T, Kohlmann A, Wieczorek L, Basso G, Kronnie GT, Bene MC, et al. Clinical utility of microarray-based gene expression profiling in the diagnosis and subclassification of leukemia: report from the International Microarray Innovations in Leukemia Study Group. J Clin Oncol. 2010;28:2529–37. 20406941 10.1200/JCO.2009.23.4732 PMC5569671

[60] Yang WS, Stockwell BR. Synthetic lethal screening identifies compounds activating iron-dependent, nonapoptotic cell death in oncogenic-RAS-harboring cancer cells. Chemistry Biol. 2008;15:234–45. 10.1016/j.chembiol.2008.02.010 PMC2683762 18355723

[61] Leo IR, Aswad L, Stahl M, Kunold E, Post F, Erkers T, et al. Integrative multi-omics and drug response profiling of childhood acute lymphoblastic leukemia cell lines. Nat Commun. 2022;13:1691. 35354797 10.1038/s41467-022-29224-5 PMC8967900

[62] Rudolph D, Impagnatiello MA, Blaukopf C, Sommer C, Gerlich DW, Roth M, et al. Efficacy and mechanism of action of volasertib, a potent and selective inhibitor of Polo-like kinases, in preclinical models of acute myeloid leukemia. Journal Pharmacol Exp therapeutics. 2015;352:579–89. 10.1124/jpet.114.221150 25576074

[63] Adachi Y, Ishikawa Y, Kiyoi H. Identification of volasertib-resistant mechanism and evaluation of combination effects with volasertib and other agents on acute myeloid leukemia. Oncotarget. 2017;8:78452–65. 29108241 10.18632/oncotarget.19632 PMC5667974

[64] Shah K, Nasimian A, Ahmed M, Al Ashiri L, Denison L, Sime W, et al. PLK1 as a cooperating partner for BCL2-mediated antiapoptotic program in leukemia. Blood cancer J. 2023;13:139. 37679323 10.1038/s41408-023-00914-7 PMC10484999

[65] Zhang C, Liu X, Jin S, Chen Y, Guo R. Ferroptosis in cancer therapy: a novel approach to reversing drug resistance. Mol Cancer. 2022;21:47. 35151318 10.1186/s12943-022-01530-y PMC8840702

[66] Dixon SJ, Olzmann JA. The cell biology of ferroptosis. Nature Rev Mol cell Biol. 2024;25:424–42. 38366038 10.1038/s41580-024-00703-5 PMC12187608

[67] Gao M, Monian P, Quadri N, Ramasamy R, Jiang X. Glutaminolysis and transferrin regulate ferroptosis. Mol Cell. 2015;59:298–308. 26166707 10.1016/j.molcel.2015.06.011 PMC4506736

[68] Dixon SJ, Stockwell BR. The role of iron and reactive oxygen species in cell death. Nature Chem Biol. 2014;10:9–17. 24346035 10.1038/nchembio.1416

[69] Tang D, Chen X, Kang R, Kroemer G. Ferroptosis: molecular mechanisms and health implications. Cell Res. 2021;31:107–25. 33268902 10.1038/s41422-020-00441-1 PMC8026611

[70] Doll S, Proneth B, Tyurina YY, Panzilius E, Kobayashi S, Ingold I, et al. ACSL4 dictates ferroptosis sensitivity by shaping cellular lipid composition. Nature Chem Biol. 2017;13:91–8. 27842070 10.1038/nchembio.2239 PMC5610546

[71] Sun X, Ou Z, Chen R, Niu X, Chen D, Kang R, et al. Activation of the p62-Keap1-NRF2 pathway protects against ferroptosis in hepatocellular carcinoma cells. Hepatology (Baltimore. Md). 2016;63:173–84. 10.1002/hep.28251 PMC4688087 26403645

[72] von Mässenhausen A, Zamora Gonzalez N, Maremonti F, Belavgeni A, Tonnus W, Meyer C, et al. Dexamethasone sensitizes to ferroptosis by glucocorticoid receptor-induced dipeptidase-1 expression and glutathione depletion. Sci Adv. 2022;8:eabl8920. 35108055 10.1126/sciadv.abl8920 PMC8809683

[73] Tontsch-Grunt U, Rudolph D, Waizenegger I, Baum A, Gerlach D, Engelhardt H, et al. Synergistic activity of BET inhibitor BI 894999 with PLK inhibitor volasertib in AML in vitro and in vivo. Cancer Lett. 2018;421:112–20. 29454094 10.1016/j.canlet.2018.02.018

[74] Boyer MW, Orchard PJ, Gorden KB, Anderson PM, McLvor RS, Blazar BR. Dependency on intercellular adhesion molecule recognition and local interleukin-2 provision in generation of an in vivo CD8+ T-cell immune response to murine myeloid leukemia. Blood. 1995;85:2498–506. 7727779

[75] Zhang L, Gajewski TF, Kline J. PD-1/PD-L1 interactions inhibit antitumor immune responses in a murine acute myeloid leukemia model. Blood. 2009;114:1545–52. 19417208 10.1182/blood-2009-03-206672 PMC2731636

[76] Choi ME, Price DR, Ryter SW, Choi AMK. Necroptosis: a crucial pathogenic mediator of human disease. JCI insight. 2019;4:e128834. 31391333 10.1172/jci.insight.128834 PMC6693822

[77] Oldenburg E, Kronberg RM, Niehoff B, Ebenhöh O, Popa O. DeepLOKI-a deep learning based approach to identify zooplankton taxa on high-resolution images from the optical plankton recorder LOKI. Front Marine Sci. 2023;10:1280510.

[78] Kleesiek J, Urban G, Hubert A, Schwarz D, Maier-Hein K, Bendszus M, et al. Deep MRI brain extraction: A 3D convolutional neural network for skull stripping. NeuroImage. 2016;129:460–9. 26808333 10.1016/j.neuroimage.2016.01.024

[79] Cicero M, Bilbily A, Colak E, Dowdell T, Gray B, Perampaladas K, et al. Training and validating a deep convolutional neural network for computer-aided detection and classification of abnormalities on frontal chest radiographs. Investigative Radiol. 2017;52:281–7. 10.1097/RLI.0000000000000341 27922974

[80] Verduijn J, Van der Meeren L, Krysko DV, Skirtach AG. Deep learning with digital holographic microscopy discriminates apoptosis and necroptosis. Cell Death Discov. 2021;7:229. 34475384 10.1038/s41420-021-00616-8 PMC8413278

[81] Sun Y, Qiao Y, Liu Y, Zhou J, Wang X, Zheng H, et al. ent-Kaurane diterpenoids induce apoptosis and ferroptosis through targeting redox resetting to overcome cisplatin resistance. Redox Biol. 2021;43:101977. 33905957 10.1016/j.redox.2021.101977 PMC8099784

[82] Lim JKM, Delaidelli A, Minaker SW, Zhang HF, Colovic M, Yang H, et al. Cystine/glutamate antiporter xCT (SLC7A11) facilitates oncogenic RAS transformation by preserving intracellular redox balance. Proc Natl Acad Sci USA. 2019;116:9433–42. 31000598 10.1073/pnas.1821323116 PMC6511045

[83] Müller F, Lim JKM, Bebber CM, Seidel E, Tishina S, Dahlhaus A, et al. Elevated FSP1 protects KRAS-mutated cells from ferroptosis during tumor initiation. Cell Death Differ. 2023;30:442–56. 36443441 10.1038/s41418-022-01096-8 PMC9950476

[84] Andreani C, Bartolacci C, Scaglioni PP. Ferroptosis: a specific vulnerability of RAS-driven cancers?. Front Oncol. 2022;12:923915. 35912247 10.3389/fonc.2022.923915 PMC9337859

[85] Luo M, Wu L, Zhang K, Wang H, Zhang T, Gutierrez L, et al. miR-137 regulates ferroptosis by targeting glutamine transporter SLC1A5 in melanoma. Cell Death Differ. 2018;25:1457–72. 29348676 10.1038/s41418-017-0053-8 PMC6113319

[86] Al Mamun Bhuyan A, Ashiqul Haque AKM, Sahu I, Cao H, Kormann MSD, Lang F. Inhibition of Suicidal Erythrocyte Death by Volasertib. Cellular Physiol Biochem : Int J Exp Cell Physiol, Biochem, Pharmacol. 2017;43:1472–86. 10.1159/000481969 29035889

[87] Van den Bossche J, Deben C, De Pauw I, Lambrechts H, Hermans C, Deschoolmeester V, et al. In vitro study of the Polo-like kinase 1 inhibitor volasertib in non-small-cell lung cancer reveals a role for the tumor suppressor p53. Molecular Oncol. 2019;13:1196–213. 10.1002/1878-0261.12477 PMC6487694 30859681

[88] Zhang L, Li XM, Shi XH, Ye K, Fu XL, Wang X, et al. Sorafenib triggers ferroptosis via inhibition of HBXIP/SCD axis in hepatocellular carcinoma. Acta pharmacologica Sin. 2023;44:622–34. 10.1038/s41401-022-00981-9 PMC9958095 36109580

[89] Lachaier E, Louandre C, Godin C, Saidak Z, Baert M, Diouf M, et al. Sorafenib induces ferroptosis in human cancer cell lines originating from different solid tumors. Anticancer Res. 2014;34:6417–22. 25368241

[90] Gao W, Wang X, Zhou Y, Wang X, Yu Y. Autophagy, ferroptosis, pyroptosis, and necroptosis in tumor immunotherapy. Signal Transduct Target Ther. 2022;7:196. 35725836 10.1038/s41392-022-01046-3 PMC9208265

[91] Friedmann Angeli JP, Krysko DV, Conrad M. Ferroptosis at the crossroads of cancer-acquired drug resistance and immune evasion. Nat Rev Cancer. 2019;19:405–14. 31101865 10.1038/s41568-019-0149-1

[92] Reda M, Ngamcherdtrakul W, Nelson MA, Siriwon N, Wang R, Zaidan HY, et al. Development of a nanoparticle-based immunotherapy targeting PD-L1 and PLK1 for lung cancer treatment. Nat Commun. 2022;13:4261. 35871223 10.1038/s41467-022-31926-9 PMC9308817

[93] Renner AG, Dos Santos C, Recher C, Bailly C, Créancier L, Kruczynski A, et al. Polo-like kinase 1 is overexpressed in acute myeloid leukemia and its inhibition preferentially targets the proliferation of leukemic cells. Blood. 2009;114:659–62. 19458358 10.1182/blood-2008-12-195867

[94] Tsykunova G, Reikvam H, Ahmed AB, Nepstad I, Gjertsen BT, Bruserud Ø. Targeting of polo-like kinases and their cross talk with Aurora kinases–possible therapeutic strategies in human acute myeloid leukemia?. Expert Opin Investig Drugs. 2012;21:587–603. 22424119 10.1517/13543784.2012.668525

[95] Döhner H, Lübbert M, Fiedler W, Fouillard L, Haaland A, Brandwein JM, et al. Randomized, phase 2 trial of low-dose cytarabine with or without volasertib in AML patients not suitable for induction therapy. Blood. 2014;124:1426–33. 25006120 10.1182/blood-2014-03-560557 PMC4148765

[96] Ottmann OG, Müller-Tidow C, Krämer A, Schlenk RF, Lübbert M, Bug G, et al. Phase I dose-escalation trial investigating volasertib as monotherapy or in combination with cytarabine in patients with relapsed/refractory acute myeloid leukaemia. British J Haematol. 2019;184:1018–21. 10.1111/bjh.15204 29882583

[97] Döhner H, Symeonidis A, Deeren D, Demeter J, Sanz MA, Anagnostopoulos A, et al. Adjunctive volasertib in patients with acute myeloid leukemia not eligible for standard induction therapy: a randomized, phase 3 Trial. HemaSphere. 2021;5:e617. 34350385 10.1097/HS9.0000000000000617 PMC8328241

[98] Wagner J, Lacher MD, Gu CJ, Leonardi C, Mannis G. A phase 2 study with volasertib for Ven-HMA relapsed/refractory acute myeloid leukemia patients guided by a predictive precision medicine platform. Blood. 2023;142:5952 (Supplement 1).

[99] Rodencal J, Kim N, He A, Li VL, Lange M, He J, et al. Sensitization of cancer cells to ferroptosis coincident with cell cycle arrest. Cell Chem Biol. 2024;31:234–48.e13. 37963466 10.1016/j.chembiol.2023.10.011 PMC10925838

[100] Gagliardi M, Cotella D, Santoro C, Corà D, Barlev NA, Piacentini M, et al. Aldo-keto reductases protect metastatic melanoma from ER stress-independent ferroptosis. Cell Death Dis. 2019;10:902. 31780644 10.1038/s41419-019-2143-7 PMC6883066

[101] Kim R, Hashimoto A, Markosyan N, Tyurin VA, Tyurina YY, Kar G, et al. Ferroptosis of tumour neutrophils causes immune suppression in cancer. Nature. 2022;612:338–46. 36385526 10.1038/s41586-022-05443-0 PMC9875862

[102] Zeng W, Zhang R, Huang P, Chen M, Chen H, Zeng X, et al. Ferroptotic neutrophils induce immunosuppression and chemoresistance in breast cancer. Cancer Res. 2025;85:477–96. 39531510 10.1158/0008-5472.CAN-24-1941 PMC11786957

[103] Tan C, Sun F, Kong T, Zhang W, Yang C, Liu C, editors. A survey on deep transfer learning. International conference on artificial neural networks. Springer; 2018.

[104] Oikonomou A, Valsecchi L, Quadri M, Watrin T, Scharov K, Procopio S, et al. High-throughput screening as a drug repurposing strategy for poor outcome subgroups of pediatric B-cell precursor Acute Lymphoblastic Leukemia. Biochemical Pharmacol. 2023;217:115809. 10.1016/j.bcp.2023.115809 37717691

