# Supplementary material for: Deep transfer learning approach for automated cell death classification reveals novel ferroptosis-inducing agents in subsets of B-ALL
Source: Cell Death Dis. 2025 May 18;16(1):396. doi: 10.1038/s41419-025-07704-y (PMC12085637; doi:10.1038/s41419-025-07704-y)
Supplement: Supplementary file 1 — supplementary figures [file 41419_2025_7704_MOESM1_ESM.docx]

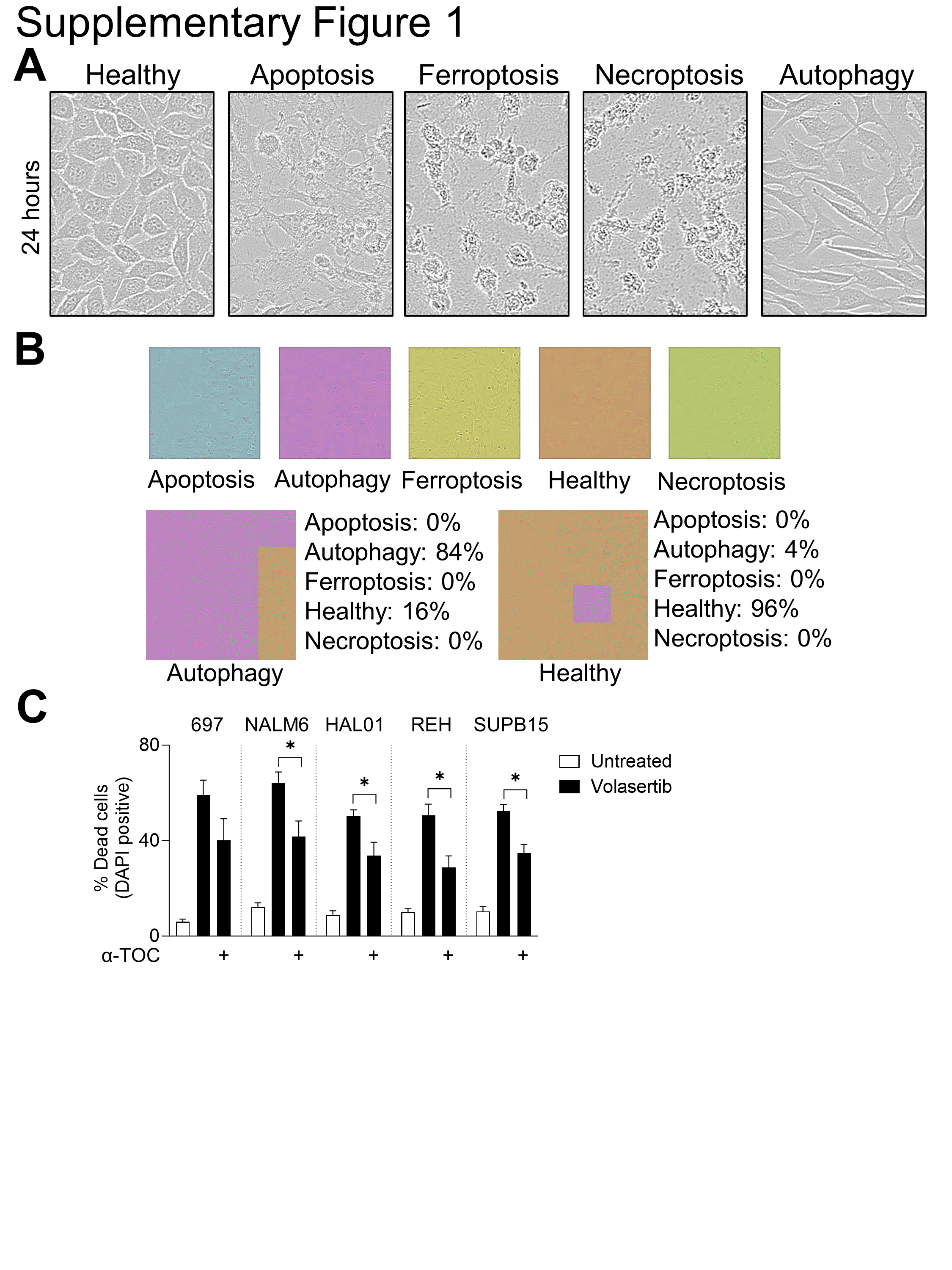


**Supplementary Figure 1**. **Deep learning algorithm correctly classifies different modes of cell death.** (A) Representative brightfield images of L929 cells treated with staurosporine for apoptosis induction, RSL3 for ferroptosis induction, zVAD and TNFα for necroptosis induction and Torin1 for autophagy induction are shown 24 hours post-treatment. Cells were treated with 1 µM staurosporine, 40 µM of zVAD, 40ng/ml of TNFα, 1µM of RSL3, 5 µM of Torin1, 10 µM of nec-1 and 5 µM Fer-1. (B) Color coded classification type of cell death in panel above and representative validation of images deep learning classification from L929 cells induced with the corresponding type of cell death in bottom panel are shown. (C) Human B-ALL cell lines were treated with 6 µM volasertib and 10 µM α-TOC for 72 hours. Viability was assessed as percentage of DAPI positive cells (n = 6). Error bars indicate SEM; *P < 0.05, as determined by 1-way ANOVA with a Dunnett’s post-hoc test.


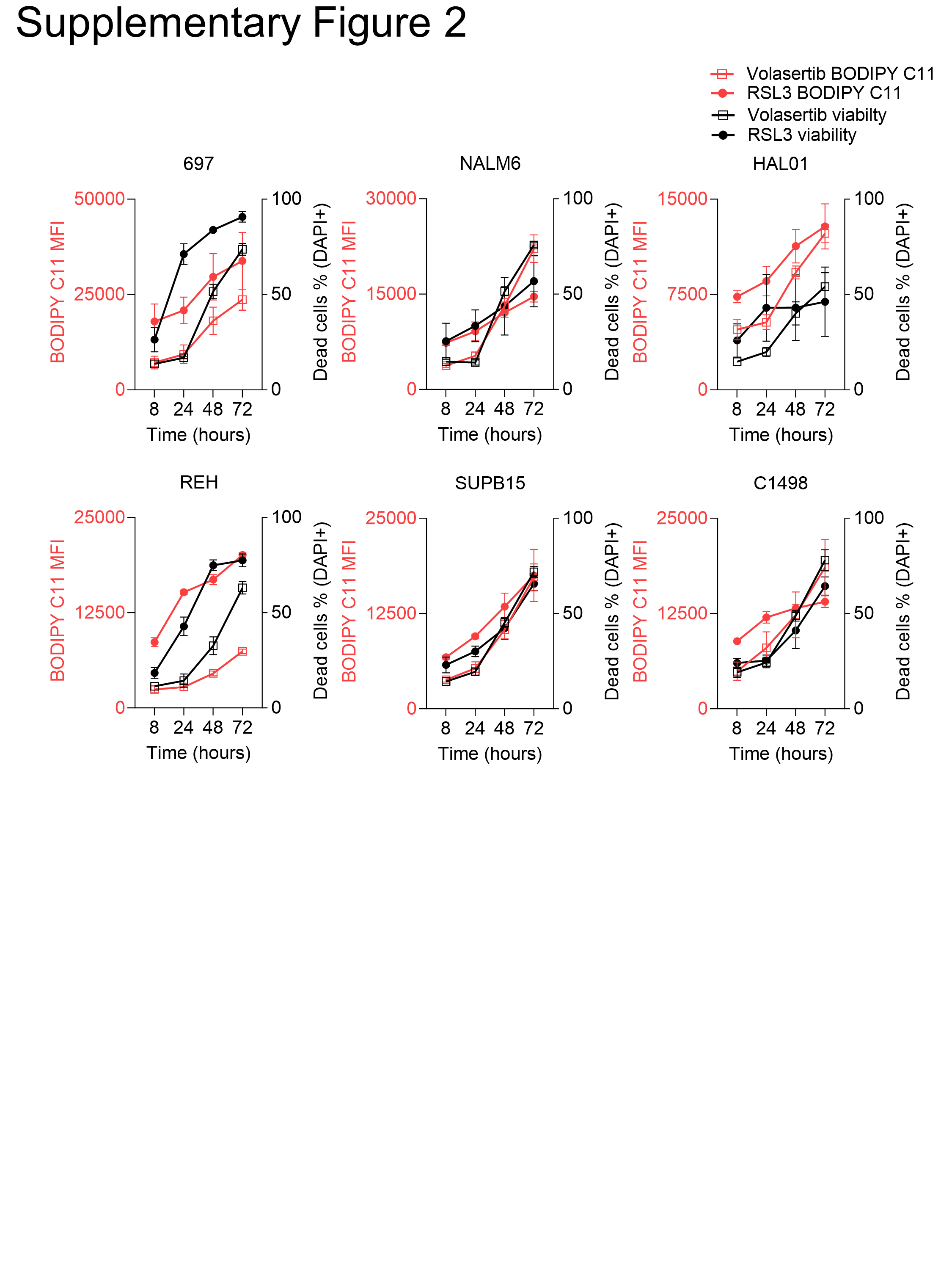


**Supplementary Figure 2. Kinetics of RSL3 and volasertib induced cell death and lipid peroxidation.** Cells were treated with 1 µM RSL3 or 6 µM volasertib and BODIPY C11 and cell viability was assessed by measuring the percentage of DAPI positive cells. The measurements were performed 8, 24, 48 and 72 hours post treatment and from the same well (n = 4-6). Error bars indicate SEM.


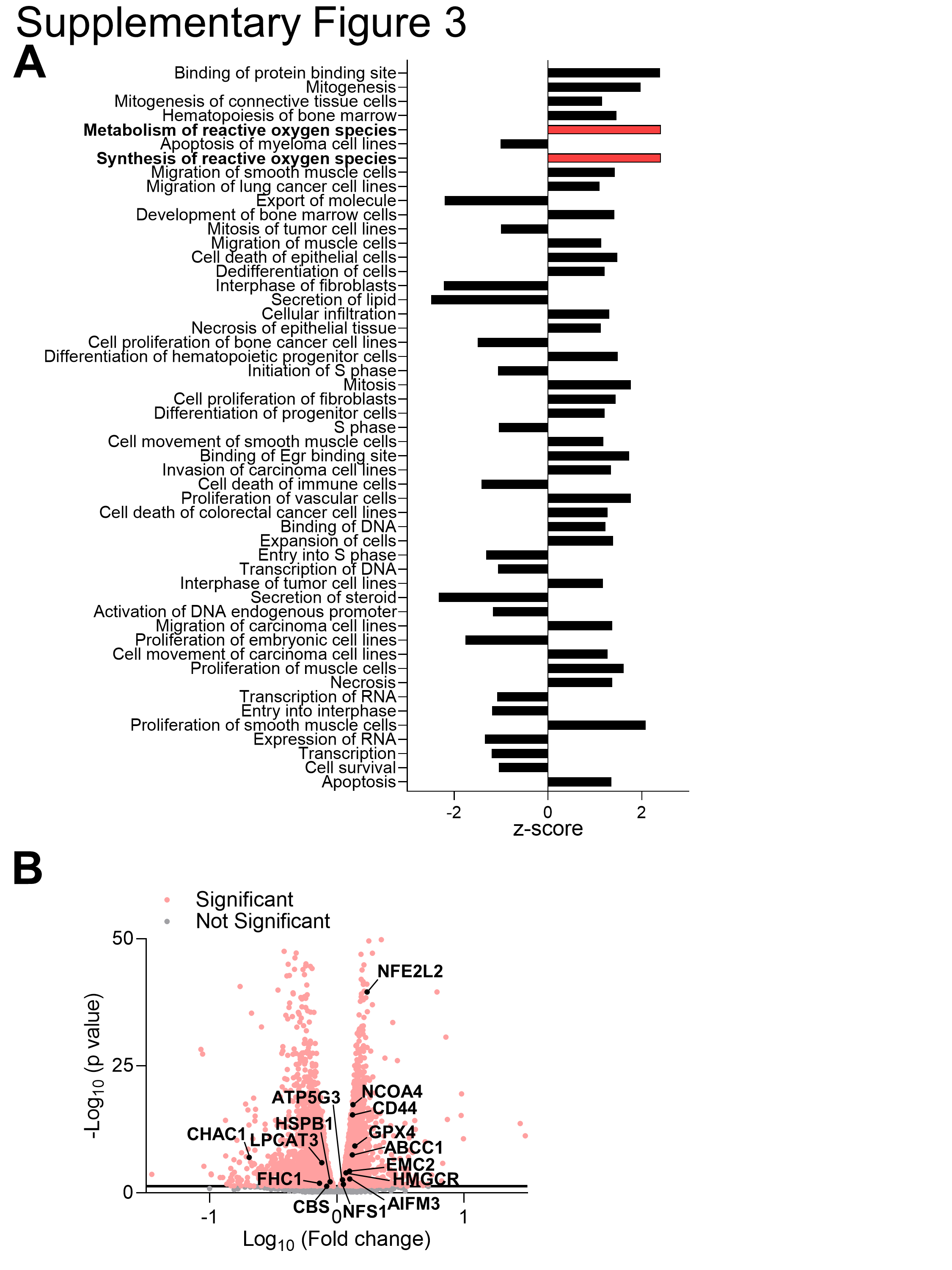


**Supplementary Figure 3**. **Volasertib induces a ferroptosis gene signature.** RNA sequencing data (GSE103068) of the human AML cell line MV-4-11B treated with volasertib were reanalyzed for (A) pathways activation, (B) volcano plot of fold change gene expression with highlighted genes involved in ferroptosis pathway.


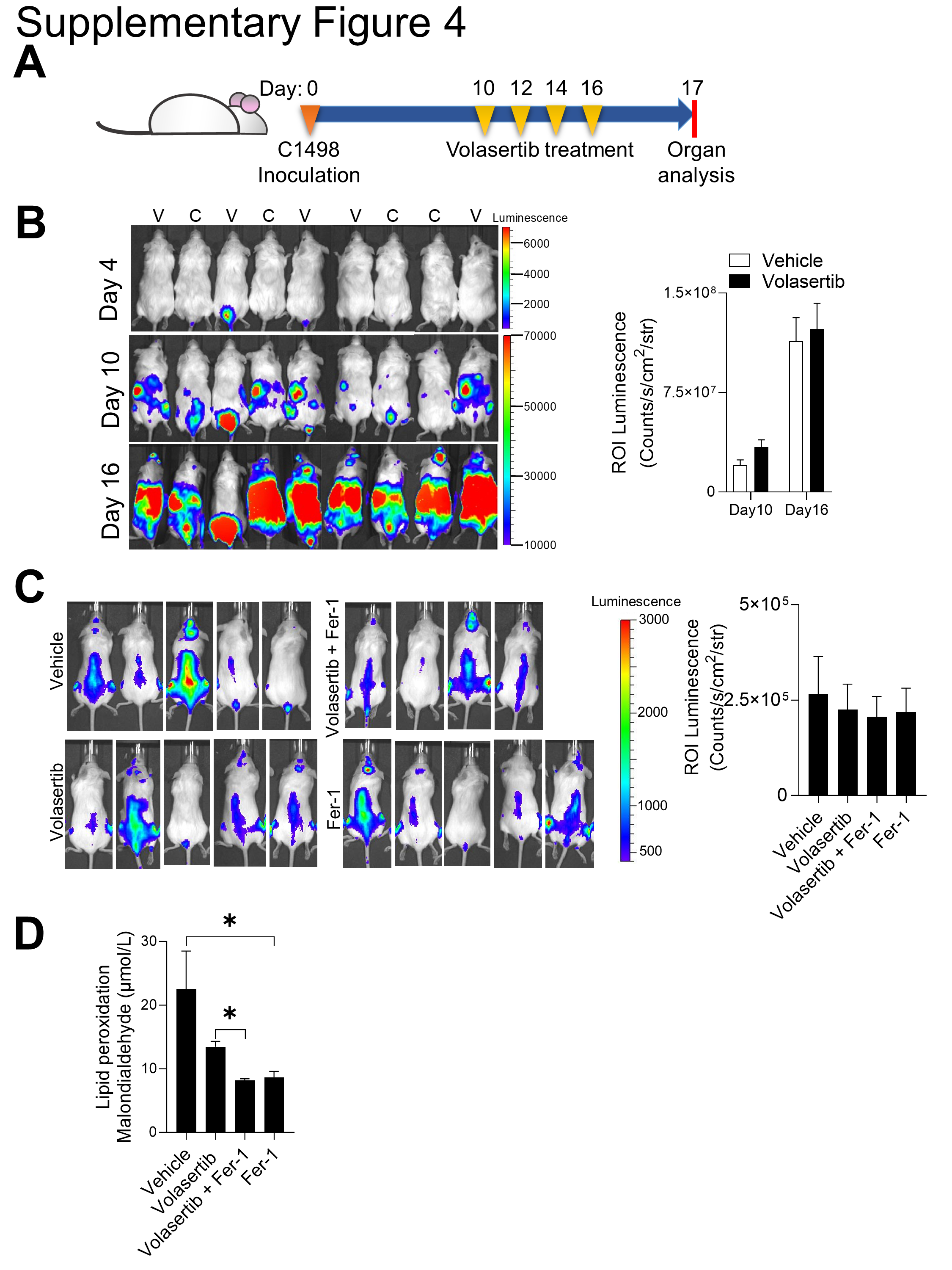


**Supplementary Figure 4.** **Volasertib treatment *in vivo*.** (A, B) NSG mice were inoculated intravenously with 5 x 10^5^ C1498-luc-GFP cells. Mice were randomized and treated with 20 mg/kg volasertib or vehicle at day 10, 12, 14 and 16 post-inoculation, as indicated in (A). Representative IVIS scan images of mice treated with volasertib or vehicle were taken and ROI signal was quantified in the right panel. (C, D) NSG mice (n = 4-5 per group) were inoculated with 10^6^ REH-luc-GFP cells and IVIS scans measuring bioluminescence were performed on day 6 post injection. Mice were randomized according to the signal into vehicle, volasertib, Fer-1 and volasertib + Fer-1. Bioluminescence was quantified in the right panel. (D) On day 16 post tumor inoculation, malondialdehyde (MDA) plasma levels were measured. Error bars indicate SEM; *P < 0.05, as determined by a 1-way ANOVA with a Dunnett’s post-hoc test.
